# Multidisciplinary Team Managements and Clinical Outcomes in Patients With Pulmonary Arterial Hypertension During the Perinatal Period

**DOI:** 10.3389/fcvm.2021.795765

**Published:** 2021-12-17

**Authors:** Tingting Shu, Panpan Feng, Xiaozhu Liu, Li Wen, Huaqiao Chen, Yunwei Chen, Wei Huang

**Affiliations:** ^1^Department of Cardiology, The First Affiliated Hospital of Chongqing Medical University, Chongqing, China; ^2^Department of Cardiology, The Second Affiliated Hospital of Chongqing Medical University, Chongqing, China

**Keywords:** pulmonary arterial hypertension, perinatal period, multidisciplinary team, pregnancy, outcomes

## Abstract

**Background:** Pulmonary arterial hypertension (PAH) patients with pregnancy have high maternal mortality. This study aimed to provide clinical evidence with multidisciplinary team (MDT) management and to evaluate the clinical outcomes in PAH patients during the perinatal period.

**Methods:** We conducted a retrospective evaluation of PAH patients pregnant at the First Affiliated Hospital of Chongqing Medical University between May 2015 and May 2021.

**Results:** Twenty-two patients (24 pregnancies) were included in this study and received MDT management, and 21 pregnancies chose to continue pregnancy with cesarean section. Nine (37.5%) were first-time pregnancies at 27.78 ± 6.16 years old, and 15 (62.5%) were multiple pregnancies at 30.73 ± 3.71 years old. The average gestational week at hospitalization and delivery were 29.38 ± 8.63 weeks and 32.37 ± 7.20 weeks, individually. Twenty-one (87.5%) pregnancies received single or combined pulmonary vasodilators. The maternal survival rate of PAH patients reached 91.7%. Fifteen (62.5%) pregnancies were complicated with severe adverse events. Patients with complicated adverse events showed lower percutaneous oxygen saturation (SpO_2_), lower albumin, lower fibrinogen, higher pulmonary artery systolic pressure (PASP), higher blood pressure, longer activated partial thromboplastin time, and longer coagulation time. Fourteen (66.7%) pregnancies with cesarean sections were prematurely delivered and 85.7% newborns who survived after the operation remained alive.

**Conclusion:** The survival rate of parturients with PAH was improved in relation to MDT and pulmonary vasodilator therapy during the perinatal period compared with previous studies. SpO_2_, albumin, PASP, blood pressure, and coagulation function should be monitored carefully in PAH patients during pregnancy.

## Introduction

Pulmonary arterial hypertension (PAH) is a severe disease with the main pathological change being elevated pulmonary vascular resistance, which often leads to right ventricular failure and death (1). The population with a high incidence includes females of childbearing age, and the first clinical manifestations may appear in pregnancy (2). Extensive physiological changes during pregnancy and delivery can exacerbate right ventricular failure in PAH patients (3), leading to high maternal mortality (between 25 and 56%) and poor neonatal outcomes (4–6). Although pregnancy has been prohibited in the European Society of Cardiology (ESC) PAH guidelines (7), some women with PAH insist on pregnancy despite the potential risks.

Advances in medical management have gradually improved the outcomes of PAH patients with pregnancy, but maternal mortality remains high (16–30%) (8, 9). The current guidelines still recommend strict contraception for PAH patients, and early termination is strongly recommended for PAH with pregnancy (7). The management of PAH patients during the perinatal period is heterogeneous in various medical centers, and the evidence of delivery modes, PAH targeted therapy, hemodynamic management, and the use of oxytocin are also unclear (7). Previous studies have suggested early clinical deterioration, severe right ventricular failure, brain natriuretic peptide elevation, and World Health Organization function class III or IV symptoms as high risk factors for maternal outcome (10–12). This study aimed to provide clinical evidence with multidisciplinary team (MDT) management and to evaluate the maternal and infant clinical outcomes in PAH patients during the perinatal period.

## Methods

### Patients

We identified PAH patients who were pregnant at the First Affiliated Hospital of Chongqing Medical University China between May 2015 and May 2021. Electronic and paper medical records of all the patients identified by the query were reviewed independently by two investigators (T. T. Shu and P. P. Feng). Inclusion in the study was predicated on a clinical diagnosis of PAH confirmed by clinical history, physical examination, right-sided heart catheterization (RHC, at the time of admission or within the preceding 5 years), or ultrasound cardiogram (UCG) (1). Patients with preexisting cardiomyopathy, left ventricular ejection fraction <40%, or mitral or aortic valve disease were excluded. Severe PAH was defined as systolic pulmonary artery pressure (PASP) ≥70 mmHg based on the highest measured value during pregnancy (9).

### Data Extraction

Data extraction included patient demographic data, including age, insurance, gestational age, expenses, etiology of PAH, comorbidities, and PAH targeted therapy before, during, and after pregnancy. The clinical baseline characteristics included clinical symptoms, blood pressure (BP), heart rate, percutaneous oxygen saturation (SpO_2_) as measured by finger oximetry, laboratory tests, UCG, and electrocardiogram reports. Perinatal data included delivery mode, the timing of delivery, type of anesthesia, medications, intraoperative bleeding, and intraoperative vital signs. Maternal and neonatal outcomes were collected, including Apgar score, neonatal weight, and referral. Severe adverse events (SAEs) were defined as death, heart failure (HF), respiratory failure (RF), and infection. The patients were divided into the SAE group and without SAE group.

### Statistical Analysis

The clinical baseline characteristics of all included pregnancies were descriptive in detail, and they were subgrouped and summarized according to underlying diseases and number of pregnancies. Data are presented as the mean ± standard deviation (SD) for parametric data, and the differences were compared between subgroups. A comparison of continuous parameters was performed using Student's *t*-test. Dichotomous variables were analyzed using χ^2^ Fisher's exact test. The difference analysis was performed by SPSS 22.0 software. A *P*-value < 0.05 was considered statistically significant.

## Results

### Baseline Characteristics

Twenty-two female patients (24 pregnancies) with PAH were identified in this study. Two patients were pregnant twice during the study. All pregnancies were natural pregnancies, and there were no medical conceptions. The baseline characteristics are shown in Table 1. Eighteen (82%) patients were diagnosed with PAH associated with congenital heart disease (PAH-CHD), three (14%) were idiopathic PAH (IPAH), and one (4%) was PAH associated with connective tissue disease (PAH-CTD). Among these patients, five were diagnosed with PAH before pregnancy, and four received pulmonary vasodilator therapy in advance. Of the 24 pregnancies, nine (37.5%) were first-time pregnancies at 27.78 ± 6.16 years old, and 15 (62.5%) were multiple pregnancies at 30.73 ± 3.71 years old. The average gestational week at hospitalization was 29.38 ± 8.63 weeks. Comorbidities included subclinical hypothyroidism (2), hypothyroidism (1), antiphospholipid syndrome (1), hysteromyoma (1), diabetes (1), colon cancer (1), and uremia (1). There was no significant difference in baseline characteristics among all subgroups according to PAH etiology and gravidity history (*P* > 0.05, Supplementary Table S1).

**Table 1:** Baseline characteristics and management of PAH patients with pregnancy.

**Patients**	**PAH age, y**	**Pregnancy age, y**	**GP history**	**Etiology**	**ES**	**NYHA class**	**BMI, kg/m^2^**	**BP, mmHg**	**HR, bpm**	**PVs therapy before pregnancy**		**PVs therapy during pregnancy**			**Comorbidity**
										**Medicine**	**Start time, y**	**Medicine**	Dose^**^	**Start time, wk**	
No. 1	30	30	G2P0	CHD (VSD)	Yes	IV	20.31	181/110	94	–	–	Treprostinil & Tadalafil	20 ng/kg/min 10 mg qd	32 32	Subclinical hypothyroidism
No. 2	29	29	G1P0	CHD (VSD)	Yes	III	17.63	102/76	107	–	–	Treprostinil	20 ng/kg/min	29	–
No. 3	31	31	G3P0	CTD	–	III–IV	21.09	92/68	79	–	–	Treprostinil & Tadalafil	20 ng/kg/min 10 mg qd	35 35	Hypothyroidism
No. 4	28	28	G1P0	IPAH	–	II–III	19.72	91/61	83	–	–	Treprostinil & Tadalafil	20 ng/kg/min 10 mg qd	14 14	–
No. 4^*^	28	30	G2P0	IPAH	–	II	22.49	104/65	80	Treprostinil, Tadalafil	28	Treprostinil & Tadalafil	20 ng/kg/min 10 mg qd	26 26	Hypothyroidism
No. 5	40	40	G1P0	CHD (ASD)	No	II	26.44	169/101	90	–	–	Treprostinil	20ng/kg/min	21	APS
No. 6	24	31	G3P0	CHD (VSD)	Yes	II–III	18.82	98/55	95	Sildenafil, Bosentan, Vardenafil, Ambrisentan, Tadalafil	25	Treprostinil & Tadalafil	20 ng/kg/min 10 mg bid	32 0	–
No. 7	30	30	G4P1	CHD (CCTGA+SV)	–	IV	25.78	136/84	100	–	–	Treprostinil	20 ng/kg/min	30	–
No. 8	25	25	G1P0	CHD (VSD)	Yes	II	20.45	121/81	100	–	–	Treprostinil	20 ng/kg/min	34	–
No. 9	36	36	G2P1	CHD (VSD)	No	II	25.81	107/63	96	–	–	Treprostinil	20 ng/kg/min	36	Hysteromyoma
No. 10	31	31	G3P1	CHD (ASD)	No	III	33.30	118/84	92	–	–	Treprostinil	20 ng/kg/min	36	–
No. 11	30	30	G3P1	CHD (ASD)	No	II	21.10	107/65	97	–	–	Treprostinil & Tadalafil	20 ng/kg/min 10 mg qd	27 27	–
No. 12	20	20	G1P0	CHD (ASD)	Yes	II	25.44	134/84	110	–	–	–	–	–	–
No. 13	22	22	G1P0	CHD (PDA)	–	III–IV	26.56	122/67	95	–	–	–	–	–	–
No. 14	18	23	G1P0	CHD (VSD)	No	II	17.30	110/52	69	–	–	–	–	–	
No. 14^*^	18	28	G2P0	CHD (VSD)	Yes	III–IV	22.96	132/77	110	Beraprost Sodium, Bosentan	23	Treprostinil & Tadalafil	20 ng/kg/min 10 mg qd	30 0	–
No. 15	38	38	G8P1	CHD (ASD)	No	II	33.59	128/57	88	–	–	–	–	–	Diabetes
No. 16	21	26	G3P0	CHD (PDA)	Yes	II	22.86	108/68	80	Bosentan, Sildenafil, Treprostinil	21	Treprostinil & Tadalafil	20 ng/kg/min 10 mg qd	33 33	–
No. 17	25	25	G3P1	IPAH	–	II–III	24.46	116/78	79	–	–	Treprostinil		31	Colon cancer
No. 18	30	30	G4P1	CHD (VSD)	No	IV	21.23	122/75	122	–	–	Sildenafil	20 mg tid	31	–
No. 19	37	37	G3P1	IPAH	–	IV	33.06	142/86	96	–	–	–	–	–	–
No. 20	32	32	G1P1	CHD (ASD)	No	III	24.84	155/101	103	–	–	–	–	–	Uremia
No. 21	28	28	G2P1	CHD (ASD)	No	I	31.20	106/57	102	–	–	–	–	–	–
No. 22	31	31	G1P0	CHD (ASD)	No	I	23.30	104/65	80	–	–	Treprostinil & Sildenafil	20 ng/kg/min 10 mg bid	12 16	–

*PAH, pulmonary arterial hypertension; y, years old; GP history, gravidity and parity history; CHD, congenital heart disease; ASD, atrial septal defect; VSD, ventricular septal defect; CTD, connective tissue disease; IPAH, idiopathic pulmonary arterial hypertension; CCTGA, corrected transposition of great arteries; SV, single ventricle; PDA, patent ductus arteriosus; ES, eisenmenger syndrome; NYHA, New York Heart Association; BMI, body mass index; BP, blood pressure; HR, heart rate; bpm, beats per minute; PVs, pulmonary vasodilators; qd, once daily; bid, twice daily; tid, three times daily; wk, week; APS, antiphospholipid syndrome.*

* *The second pregnancy during the study period.*

** *Treprostinil was titrated gradually from a low dose to a maintenance dose according to the instructions*.

Clinical symptoms on admission are shown in Supplementary Table S2, including shortness of breath (20), decreased exercise tolerance (19), palpitation (5), cyanosis (5), dyspnea in the semireclining position (4), acropachy (2), hemoptysis (2), edema (2), thorcalgia (1), syncope (1), and asymptomatic (2). Twelve (50%) of the 24 pregnancies had electrocardiographic abnormalities on admission, including sinus tachycardia (8), right bundle branch block (5), and left anterior fascicular block (2). Eleven patients had PAH-CHD (57.9%), and one had PAH-CTD. All patients underwent UCG examination after admission, and only 7 (30%) underwent RHC within the preceding 5 years. The echocardiographic or RHC parameters at the time of diagnosis of included PAH patients are shown in Supplementary Table S3.

The hospital admission indicators before termination of pregnancy or delivery are shown in Table 2. The average value of SpO_2_ was 92.54% ± 9.4%, PASP was 73.74 ± 27.35 mmHg, and Barthel index was 90.00 ± 15.11 (Supplementary Table S4). The SpO_2_ level in two patients was <70% in the non-oxygenated state. The difference in the Barthel index in disease subgroups was significant (*P* < 0.05), and the other indicators did not differ among the subgroups.

**Table 2:** Examination indicators and echocardiographic value before termination or delivery in PAH patients with pregnancy.

**Patients**	**SpO_2_, %**	**HB, g/L**	**PLT, × 10^9^/L**	**PT, s**	**APTT, s**	**Fbg, g/L**	**ALB, g/L**	**BNP, pg/ml**	**NT-proBNP, ng/L**	**Braden score**	**Barthel index**	**Echocardiographic value**						
												**PASP, mmHg**	**EF, %**	**LA diameter, mm**	**LV diameter, mm**	**RA diameter, mm**	**RV diameter, mm**	**PA diameter, mm**
No. 1	69	190	58	11.1	38.3	2.48	25	125	–	13	90	139.1	60	26	50	43	22	31
No. 2	94	138	161	11.4	28	3.66	33	–	–	15	85	61	56	25	41	32	22	30
No. 3	99	126	212	10.9	28.0	5.0	30	–	863	18	30	103	69	27	39	45	28	34
No. 4	99	122	199	12.4	34.2	3.67	35	65.1	–	13	100	96.1	62	24	42	34	25	27
No. 4^*^	96	106	151	10.7	27.3	4.56	36	–	116	16	95	79	68	29	43	42	28	43
No. 5	98	111	198	11.2	24.5	3.27	38	379	<1	17	80	71.5	67	42	42	30	27	**–**
No. 6	83	87	160	12	37.9	2.46	38	–	257	14	95	58	67	32	47	66	30	32
No. 7	65	125	194	12.4	34.5	3.67	28	–	4,249	15	90	93^#^	–	49	–	51	–	27
No. 8	91	141	138	10.2	29.1	4.36	43	420	–	18	90	123	59	35	42	42	24	25
No. 9	98	122	256	10.2	21.5	4.68	36	–	17	21	95	60.8	67	24	47	28	18	**–**
No. 10	99	106	234	11.7	28.7	4.82	36	–	1,360	15	90	76	46	44	70	42	26	20
No. 11	98	110	216	9.8	29.7	4.2	30	<5	–	13	95	50	70	30	42	43	33	29
No. 12	97	122	224	11.9	24.3	4.35	33	–	206	13	100	60	65	34	37	60	39	35
No. 13	99	96	180	11.6	24.7	4.54	34	70	–	13	95	56	62	32	43	49	29	31
No. 14	99	152	128	13.8	40	2.9	40	–	64	13	100	61	72	24	41	30	17	25
No. 14^*^	82	153	102	10.9	30.7	4.33	38	13.7	–	14	100	66	63	24	44	33	20	32
No. 15	99	128	191	11.1	23.2	4.35	35	–	68	13	95	50.2	68	32	45	46	37	**–**
No. 16	91	180	130	10.9	30.3	3.97	39	17.5	–	13	100	81	63	31	49	44	24	37
No. 17	86	25	895	15	23.6	5.08	22	–	1,510	20	65	50.3	67	37	57	36	22	23
No. 18	90	88	180	17.1	44.4	2.87	24	882	2,540	14	90	127	58	45	60	46	26	45
No. 19	98	110	279	10.8	23.5	3.86	27	–	1,660	15	95	64.5	68	31	49	38	20	**–**
No. 20	97	86	267	12.2	28	5.22	37	–	>35,000	14	85	38	51	36	62	34	20	**–**
No. 21	97	125	290	10.4	27.7	4.19	36	–	14	13	100	35	59	31	45	43	31	28
No. 22	97	115	171	10.7	27.5	3.29	38	–	147	14	100	70.2	67	31	37	32	46	24

*PAH, pulmonary arterial hypertension; SpO_2_, percutaneous oxygen saturation; HB, hemoglobin; PLT, platelet; PT, coagulation time; APTT, activated partial thromboplastin time; Fbg, fibrinogen; ALB, albumin; BNP, B-type natriuretic peptide; NT-proBNP, N terminal pro B-type natriuretic peptide; PASP, pulmonary artery systolic pressure; EF, left ventricular ejection fraction; LA, left atrium; LV, left ventricle; RA, right atrium; RV, right ventricle; PA, main pulmonary artery.*

* *The second pregnancy during the study period.*

# *Tricuspid valve pressure difference*.

### Managements and Delivery

The included patients received medical care from a MDT consisting of PAH physician experts, obstetricians, intensive care unit (ICU) physicians, anesthesiologists, neonatologists, and specialized ICU nurses. All 24 pregnancies were notified of high risk after admission and recommended termination of pregnancy. The average gestational week of termination or delivery was 32.37 ± 7.20 weeks. A total of three patients chose to terminate their pregnancy through medical induction within 20 weeks of gestation (Table 3). Twenty-one cases chose to continue their pregnancy for cesarean section, including two patients with intrauterine stillbirth at 31.6 and 24.9 weeks of gestation.

**Table 3:** Intraoperative management of pregnancy termination in PAH patients with pregnancy.

**Patients**	**GW of delivery, wk**	**Delivery mode**	**Anesthesia**	**PFDW**	**Uterine contraction**	**Oxytocin, IU**	**PB, ml**	**Amniotic fluid, ml**	**Ligation of oviduct**	**Length of operation, min**	**The moment of fetus retrieval**				
											**T, **°**C**	**RR, bpm**	**HR, bpm**	**BP, mmHg**	**SpO_2_, %**
No. 1	37	Cesarean section	TGA	Natural	Strong	20	500	400	No	55	36.5	12	86	162/88	81
No. 2	34.4	Cesarean section	EA	Natural	Strong	5	500	350	Yes	120	36.5	23	98	104/57	97
No. 3	37	Cesarean section	SA + NTGA	Natural	Strong	10	300	350	Yes	115	36.5	40	94	105/61	100
No. 4	14.6	Medical induction	**–**	Uterine curettage	**–**	**–**	110	**–**	**–**	–	–	**–**	**–**	–	–
No. 4^*^	35.3	Cesarean section	EA	Natural	Strong	0	200	500	Yes	125	35.5	16	77	120/58	99
No. 5	21.1	Medical induction	**–**	Natural	**–**	**–**	300	**–**	**–**	–	–	**–**	**–**	–	–
No. 6	32.3	Cesarean section	EA	Manual	Strong	15	500	500	Yes	130	36.4	20	90	141/70	100
No. 7	31.3	Cesarean section	TGA	Manual	Weak	0	600	1,000	Yes	81	36	12	90	118/73	93
No. 8	35	Cesarean section	TGA	Natural	Weak	0	500	600	Yes	47	36.5	12	73	79/48	94
No. 9	37	Cesarean section	EA	Natural	Weak	0	300	600	Yes	85	36.3	14	82	126/67	100
No. 10	37.4	Cesarean section	EA	Natural	Poor	0	100	100	No	70	36.5	28	109	112/71	99
No. 11	36.9	Cesarean section	EA	Natural	Strong	10	200	400	Yes	70	36	21	102	118/75	100
No. 12	37.9	Cesarean section	EA	Natural	Strong	5	300	700	Yes	110	36.3	15	104	146/85	100
No. 13	36.3	Cesarean section	EA	Natural	Strong	10	200	500	No	70	37.2	17	119	102/61	100
No. 14	11.3	Medical induction	**–**	Uterine curettage	**–**	**–**	50	**–**	**–**	–	–	**–**	**–**	–	–
No. 14^*^	33.9	Cesarean section	EA	Natural	Weak	5	300	500	Yes	80	36	26	83	165/81	92
No. 15	36.3	Cesarean section	EA	Natural	Strong	10	500	500	Yes	220	36.5	25	91	135/71	100
No. 16	34	Cesarean section	TGA	Manual	Strong	10	400	500	Yes	64	36.5	12	70	125/72	99
No. 17	31.6	Cesarean section	TGA	Natural	Strong	20	200	500	No	40	36.8	13	80	113/68	100
No. 18	32.7	Cesarean section	EA	Natural	Poor	20	750	600	No	70	36.6	21	133	163/95	100
No. 19	24.9	Cesarean section	SA + NTGA	Natural	Strong	20	750	300	No	125	36.5	24	76	153/76	100
No. 20	33.9	Cesarean section	SA + NTGA	Natural	Strong	10	200	400	No	90	37.3	27	108	161/91	99
No. 21	37.4	Cesarean section	TGA	Natural	Weak	20	300	1,200	No	38	36.5	12	115	105/60	99
No. 22	37.4	Cesarean section	EA	Natural	Weak	5	200	600	No	91	35.7	22	90	147/77	100

*PAH, pulmonary arterial hypertension; GW, gestational week; wk, week; TGA, general anesthesia with tracheal intubation; SA + NTGA, spinal anesthesia combined with non–tracheal intubation of general anesthesia; EA, epidural anesthesia; PFDW, placenta and fetal membrane delivery way; IU, international unit; PB, peroperative bleeding; T, temperature; RR, respiratory rate; bpm, beats per minute; HR, heart rate; BP, blood pressure; SpO_2_, percutaneous oxygen saturation.*

* *The second pregnancy during the study period*.

**Figure 1:**
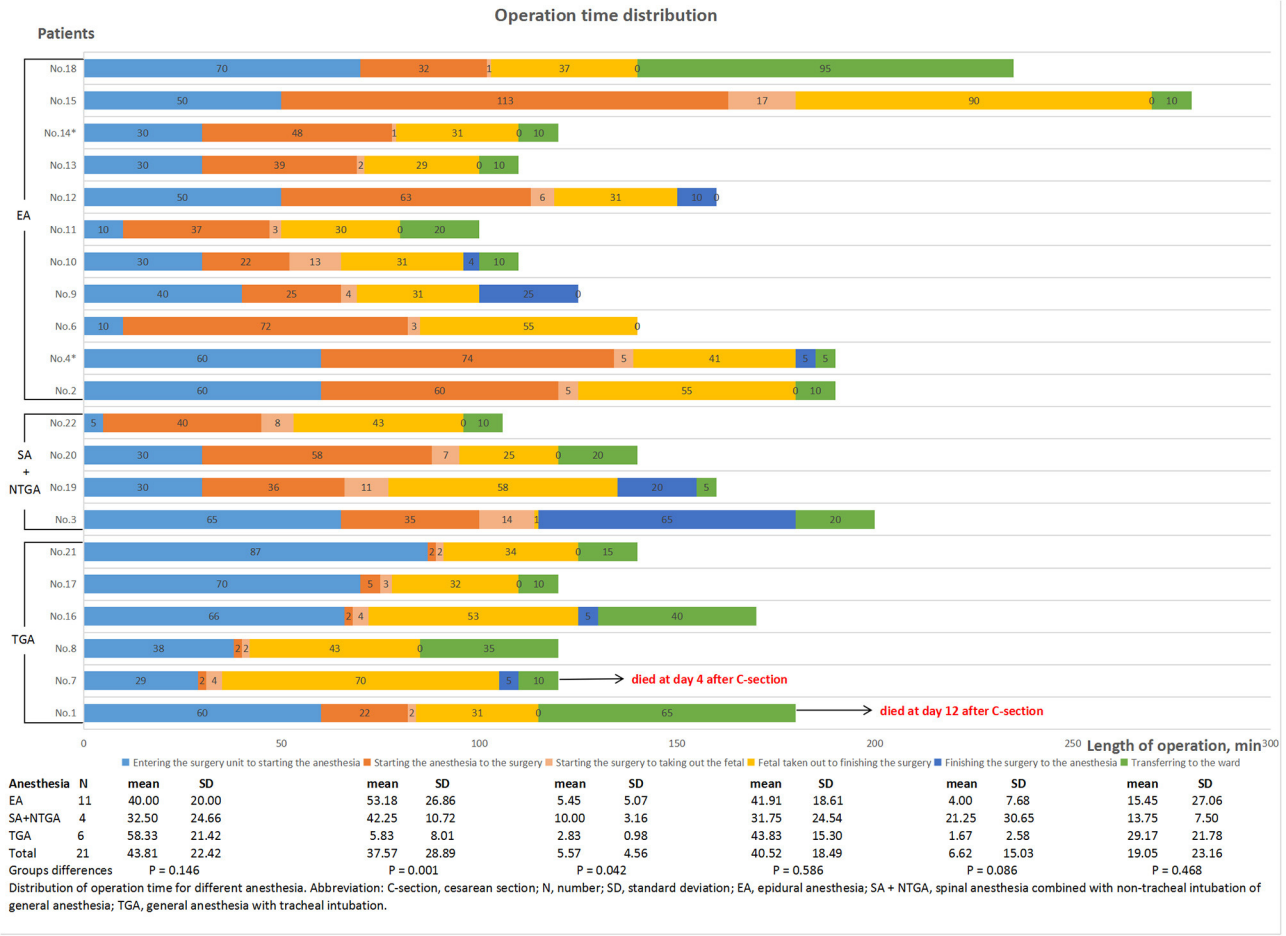
Distribution of operation time for different anesthesia conditions.

These patients received different anesthesia methods, including six patients with general anesthesia with tracheal intubation (TGA), 11 with epidural anesthesia, and four with spinal anesthesia combined with non-tracheal intubation of general anesthesia (NTGA). During cesarean section, 12 (57.1%) patients underwent ligation of the oviduct. At the moment of fetal retrieval, the SpO_2_ of four patients was lower than 95%, and one patient had complicated hypotension. The mean perioperative bleeding of these patients was 344.17 ± 193.01 ml. The distribution of operation time, intraoperative vital sign monitoring, and medication for each patient are shown in Figure 1 and Supplementary Figures S1–S21. The distribution of the operation time from starting anesthesia to surgery and from starting surgery to removing the fetus among the groups treated with various anesthesia methods was statistically significant (*P* = 0.001 and *P* = 0.042, respectively, Figure 1). Of the 21 patients undergoing cesarean section, 20 (95.2%) were transferred to the ICU immediately after surgery and received intensive monitoring of vital signs.

All PAH patients after pregnancy were reminded to receive adequate rest and nutrition. General medications were administered to 24 patients: Before delivery, antibiotics (8), diuretics (8), calcium channel blockers (CCB, 3) were used to control hypertension, and heparin (2). During delivery, antibiotics (18) and intrauterine injection of oxytocin (16), noradrenaline (9), dopamine (3), nitroglycerin (3), adrenaline (1), and dobutamine hydrochloride (1) were administered. After delivery, antibiotics (24), diuretics (10), noradrenaline (3), dopamine (2), heparin (2), CCB (2), angiotensin-converting enzyme inhibitor (1), and angiotensin receptor blocker (1) were administered.

### Pulmonary Vasodilators

Overall, eight patients received pulmonary vasodilators throughout the perinatal period, while three did not receive any PAH-targeted therapies (Tables 1, 4, and Supplementary Figures S1–S21). There were 21 (87.5%) pregnancies receiving PAH-targeted therapy: 17 (70.8%) of them received pulmonary vasodilators before operation, of which seven received treprostinil alone, one received sildenafil alone, eight received treprostinil combined with tadalafil, and one received treprostinil combined with sildenafil; 10 (41.7%) received treprostinil intraoperatively; and 19 (79.2%) received post-operatively, of which seven pregnancies received monotherapy (treprostinil or sildenafil) and 12 were treated with a combination of pulmonary vasodilators.

**Table 4:** Post-operative management and outcome at discharge of PAH patients with pregnancy.

**Patients**	**Maternal outcome at hospital discharge**				**ICU stay, d**	**Hospital stay, d**	**Hospitalization expenditure, million**	**Proportion of drug expenditure, %**	**Medical insurance**	**PVs therapy after delivery**		**Present status**	**Time after discharge**
	**Survival status**	**HF**	**RF**	**Infection**						**Medicine**	**Dose**		
No. 1	Deceased	Yes	Yes	Yes	6	20	8.18	13.4	URBMI	Treprostinil	20 ng/kg/min	–	–
No. 2	Alive	Yes	No	No	4	32	8.82	5.85	URBMI	Treprostinil & Sildenafil	20 ng/kg/min 25 mg tid	Alive	4.5 y
No. 3	Alive	No	Yes	Yes	13	35	16.67	19.64	URBMI	Treprostinil & Tadalafil & Ambrisentan	20 ng/kg/min 10 mg qd 2.5 mg qd	Alive	4 y
No. 4	Alive	No	No	No	0	15	2.66	0.82	UEBMI	Treprostinil & Tadalafil	20 ng/kg/min 10 mg qd	Alive	4 y
No. 4^*^	Alive	No	No	No	1	13	2.51	5.1	UEBMI	Treprostinil & Tadalafil	20 ng/kg/min 20 mg qd	Alive	2 y
No. 5	Alive	Yes	No	No	2	16	1.59	8.56	URBMI	Treprostinil & Sildenafil & Bosentan	20 ng/kg/min 25 mg tid 62.5 mg tid	Alive	3.5 y
No. 6	Alive	Yes	Yes	Yes	0	24	5.27	16.13	UEBMI	Treprostinil & Tadalafil	20 ng/kg/min 20 mg qd	Alive	4 y
No. 7	Deceased	Yes	Yes	Yes	13	18	4.93	19.11	URBMI	Treprostinil	20 ng/kg/min	–	–
No. 8	Alive	No	Yes	Yes	11	15	4.14	12.41	URBMI	Treprostinil	20 ng/kg/min	Alive	2 y
No. 9	Alive	No	No	Yes	1	10	2.04	9.44	Self–supporting	Treprostinil	20 ng/kg/min	Alive	2 y
No. 10	Alive	No	No	No	6	16	2.98	15.63	Self–supporting	–	–	Alive	2 y
No. 11	Alive	No	No	No	1	15	2.34	9.83	Self–supporting	Treprostinil & Tadalafil	20 ng/kg/min 10 mg qd	Alive	2 y
No. 12	Alive	Yes	No	No	4	16	2.50	9.07	URBMI	Treprostinil & Sildenafil	20 ng/kg/min 25 mg tid	Alive	14 m
No. 13	Alive	No	No	No	3	10	2.30	19.9	URBMI	Treprostinil & Bosentan	20 ng/kg/min 62.5 mg bid	Alive	14 m
No. 14	Alive	Yes	No	Yes	0	8	0.73	8.82	Self–supporting	–	–	Alive	5 y
No. 14^*^	Alive	No	No	No	1	32	2.79	5.41	URBMI	Treprostinil & Tadalafil & Macitentan & Beraprost Sodium	20 ng/kg/min 10 mg qd 10 mg qd 40 ug tid	Alive	13.5 m
No. 15	Alive	No	No	No	1	7	1.64	10.18	UEBMI	Treprostinil	20 ng/kg/min	Alive	13 m
No. 16	Alive	Yes	No	Yes	1	18	2.50	8.45	NRCMS	Treprostinil & Tadalafil	20 ng/kg/min 10 mg qd	Alive	18.5 m
No. 17	Alive	No	No	Yes	3	12	4.74	23.87	URBMI	Treprostinil	20 ng/kg/min	Alive	5.5 m
No. 18	Alive	Yes	Yes	Yes	4	17	4.62	18.41	URBMI	Sildenafil	20 mg tid	Alive	16.5 m
No. 19	Alive	Yes	No	No	3	11	2.49	7.39	UEBMI	–	–	Alive	4.5 m
No. 20	Alive	Yes	No	No	1	13	2.87	10.03	URBMI	–	–	Alive	5.5 m
No. 21	Alive	No	No	No	0	6	1.85	15.56	UEBMI	–	–	Alive	17.5 m
No. 22	Alive	No	No	No	2	5	1.59	6.5	URBMI	Treprostinil	20 ng/kg/min	Alive	1.5 m

*PAH, pulmonary arterial hypertension; HF, heart failure; RF, respiratory failure; ICU, intensive care unit; URBMI, urban residents basic medical insurance; UEBMI, urban employee basic medical insurance; NRCMS, new rural cooperative medical scheme; PVs, pulmonary vasodilators; qd, once daily; bid, twice daily; tid, three times daily; y, year; m, month.*

* *The second pregnancy during the study period*.

### Maternal Outcomes

No deaths occurred during pregnancy. The maternal survival rate of PAH patients who became pregnant in the present study was 91.7%. Two (8.3%) patients died during the early post-partum period (4 and 12 days after delivery, individually). Both of the deceased patients had PAH-CHD, severe PAH, right heart failure, and multiple pregnancies. Their SpO_2_ with oxygen was <70% under room air before surgery and <95% at the moment of fetal retrieval, and they both received cesarean section under TGA. One patient had an atrial septal defect (ASD), and the other patient had corrected transposition of great arteries combined with a single ventricle. Other patients discharged from the hospital remained alive to the point of presentation of this study. The follow-up period for discharge ranged from 1.5 months to 4.5 years (median 2 years).

Fifteen (62.5%) pregnancies were complicated with SAE, and their RV diameter was larger than those without SAE (30.56 ± 7.57 mm vs. 24.21 ± 5.67 mm, *P* < 0.032, Supplementary Table S5). Eleven (45.8%) pregnancies were complicated HF, six (25%) suffered RF, and 10 (41.7%) suffered post-operative infections. Compared with the subgroups without adverse events, deceased patients had a lower admitted SpO_2_, lower albumin (ALB), higher PASP, and higher BP (*P* < 0.05, Table 4 and Supplementary Table S5), patients complicated HF showed a longer activated partial thromboplastin time (APTT) and lower fibrinogen (Fbg), patients complicated with RF showed a lower SpO_2_, longer APTT, lower Barthel index, higher PASP, and higher BP. Patients complicated with post-operative infections had a lower SpO_2_, longer coagulation time, longer APTT, and higher PASP. TGA was significantly related to death and infection (Supplementary Table S5).

The vital signs of 21 patients undergoing cesarean section were closely monitored during the operation (Supplementary Figures S1–S21). Thirteen (61.9%) pregnancies had SAE, including two pregnancies (9.5%) that died, nine (42.9%) complicated with HF, six (28.6%) that suffered RF, and nine (42.9%) that suffered post-operative infections (Table 4). Compared with the subgroups without corresponding adverse events, lower SpO_2_ at the time of fetal removal was related to death and RF (*P* = 0.00 and 0.049, respectively, Supplementary Table S6), and higher systolic and diastolic BP were related to HF (*P* = 0.032 and 0.018, respectively, Supplementary Table S6). Other vital signs were not significantly different in the subgroups with or without adverse events.

The average hospital stay for 24 pregnancies was 16 ± 8.0 days, of which the ICU stay period was 3.4 ± 3.9 days (Table 4). Compared with childbirth without PAH, the hospital stay of PAH women was significantly longer (13). The average expenditure during hospitalization before reimbursement was ¥ 38645.6 ± 33660.5, which was 10 times the expanses without PAH (¥ 3824.50) (14). The medical insurance type in 14 (58.3%) pregnancies was urban resident basic medical insurance (URBMI, reimbursement ratio 20%), six (25%) were urban employee basic medical insurance (UEBMI, reimbursement ratio 75%), one (4.2%) was a new rural cooperative medical scheme (NRCMS, reimbursement ratio 20%), and three (12.5%) had not purchased any medical insurance were paying for medical care at their own expense.

### Fetal/Neonatal Outcomes

Eighteen (85.7%) newborns who survived after the operation remained alive (Table 5). Eight (44.4%) newborns were transferred to the neonatology department immediately after birth, and the families of the other 10 (55.6%) newborns refused section transfer. In the 21 cesarean sections, 14 (66.7%) pregnancies were prematurely delivered.

**Table 5:** Status of the fetus and the newborn after delivery.

**Patients**	**Fetal status**		**Neonate**				**Apgar score**			**Transfer to neonatology**
	**IUGR**	**FGR**	**Survival status**	**Birth weight, g**	**Gender**	**Premature**	**1 min**	**5 min**	**10 min**	
No. 1	Yes	Yes	Alive	1,190	Male	Full-term	5	5	6	No
No. 2	No	No	Alive	UN	Male	Pre-term	9	10	10	No
No. 3	Yes	No	Alive	UN	Female	Full-term	9	9	10	Yes
No. 4^*^	No	No	Alive	UN	Male	Pre-term	9	10	10	Yes
No. 6	No	No	Alive	1,800	Male	Pre-term	8	9	9	Yes
No. 7	Yes	Yes	Deceased^**^	UN	Female	Pre-term	5	8	8	No^**^
No. 8	Yes	No	Alive	UN	Female	Pre-term	6	8	9	No
No. 9	No	No	Alive	UN	Female	Full-term	10	10	10	Yes
No. 10	No	No	Alive	2,850	Female	Full-term	9	10	10	No
No. 11	No	No	Alive	2,860	Female	Pre-term	9	10	10	No
No. 12	No	No	Alive	3,605	Male	Full-term	9	10	10	No
No. 13	No	No	Alive	2,525	Female	Pre-term	10	10	10	No
No. 14^*^	Yes	No	Alive	UN	Male	Pre-term	9	10	10	Yes
No. 15	No	No	Alive	2,755	Male	Pre-term	10	10	10	No
No. 16	Yes	No	Alive	1,725	Male	Pre-term	9	10	10	Yes
No. 17	Yes	Yes	Stillborn^***^	1,000	Female	Pre-term	UN	UN	UN	No^***^
No. 18	No	No	Alive	UN	Male	Pre-term	10	10	10	Yes
No. 19	No	No	Stillborn^***^	UN	Male	Pre-term	UN	UN	UN	No^***^
No. 20	Yes	No	Alive	1,885	Female	Pre-term	9	9	10	Yes
No. 21	No	No	Alive	2,600	Male	Full-term	5	7	8	Yes
No. 22	Yes	No	Alive	2,465	Male	Full-term	9	10	10	No

* *The second pregnancy during the study period.*

** *The newborn died on the second day after birth.*

*** *The two fetuses were dead before the cesarean section.*

*IUGR, fetal in utero distress; FGR, fetal growth restriction; UN, unknown*.

## Discussion

This study observed a high survival rate in parturients and neonates with PAH pregnancy, which may be related to intensive management of MDT and pulmonary vasodilator therapy during the perinatal period. However, the incidence of maternal complications was still high, causing long hospital stays and high expenditures. PAH patients had high risks of poor outcomes during the perinatal period, especially in the early post-partum period. The monitoring time by MDT for PAH patients with pregnancy started slightly late in this center. The present study observed a strong association between SAE and low SpO_2_ and ALB, high PASP, increased right heart diameter and BP, and severe coagulopathy.

### Pulmonary Vasodilator Therapy

With the development of PAH-targeted therapies such as prostacyclins, phosphodiesterase inhibitors 5 (PDE5i), and endothelin receptor antagonists (ERAs), PAH has been well-controlled (1). In this study, most patients used single (prostacyclins or PDE5is) or combined (prostacyclins combined with PDE5is) pulmonary vasodilator therapy during the perinatal period, and the maternal survival rate reached 91.7%. Perinatal PAH targeted therapy was beneficial to the maternal outcomes of PAH (1, 15). However, there is a lack of clinical evidence for the recommended dose of pulmonary vasodilators for PAH patients during pregnancy. In the present study, treprostinil was initiated at 1.25 ng/kg/min and titrated by 2.5 ng/kg/min every 6 h to a final dose of 20 ng/kg/min during the pre- and post-operative period (16) which was lower than the effective dose (40–60 ng/kg/min) routinely used for non-pregnant PAH patients (17, 18). Mainly due to it not being reimbursed in China and high cost (¥ 9,800/20 mg), it was administered at 5 ng/kg/min to 50 ng/kg/min during the operative period. Treprostinil, tadanafil, and sildenafil are not included in medical insurance in China. These drugs were purchased outside the hospital, and this cost was not included in the hospitalization expenses. Studies have shown that ERAs are teratogenic (3) and should be suspended during pregnancy and used in combination after delivery. Due to the physiological changes and complications that occur during pregnancy, it is vital to monitor patients carefully and make dose adjustments as necessary throughout pregnancy and delivery (7).

### Clinical Outcomes

Previous systematic overviews and selected case series have reported a high mortality rate (12–56%) in PAH women pregnant (5, 19). However, little is known about the risk factors related to the adverse events of perinatal PAH. In the present study, the incidence of SAE was high (62.5%), and two patients with congenital heart disease died in the early perinatal period. Both of them were in serious hypoxemia (mean SpO_2_ 67% under room air) when admitted to the hospital and did not receive pulmonary targeted vasodilator treatment before pregnancy. The two deceased patients received oxygen inhalation and targeted PAH therapy after admission, but the level of SpO_2_ was still <95% with 5 L/min oxygen supplementation at the moment of fetal retrieval. Furthermore, this study found that patients with low admitted SpO_2_ had more intraoperative blood loss, which may be related to the weak contraction of the uterine muscles under hypoxia (20). Long-term refractory hypoxia might be related to an increased risk of death (21). Admitted SpO_2_, low ALB, elevated PASP and BP were significantly related to death in this study. PAH patients with abnormalities above during pregnancy should be strongly recommended to prohibit or terminate pregnancy.

All patients undergoing cesarean section were closely monitored for intraoperative vital signs. This study found that patients with adverse events presented with lower SpO_2_ and higher BP at the moment of fetal retrieval. PAH patients with the above abnormalities during surgery should be further vigilant about poor outcomes after delivery. These potential risk factors need to be confirmed in prospective studies in the future.

Pregnant women are accompanied by physiological changes with increased cardiac output. However, the diseased pulmonary vascular system of PAH patients cannot withstand increased cardiac output, resulting in strain and dilation of the right ventricle and ultimately decompensation (3, 12). This study found that severely increased PASP was prone to death, which was consistent with the risk factors proposed in the current guidelines (1, 7). The present study also showed that a higher PASP was significantly related to the occurrence of RF and infection. Eclampsia is one of the dangerous complications of pregnant women (22). There were four patients with hypertension at admission, and elevated BP was associated with death and/or HF. BP at the moment of fetal retrieval in eight patients was higher than the normal level, except one was lower. PAH patients during pregnancy might be more sensitive to BP fluctuations. Therefore, more close monitoring of BP in patients with PAH during pregnancy is necessary. Pregnant women are at risk of hypercoagulability, especially fatal pulmonary embolism (23). Antithrombosis is traditionally used in pregnancies during the perinatal period. More detailed studies on the dosage and time of antithrombotic treatment are needed.

### MDT Management

**Figure 2:**
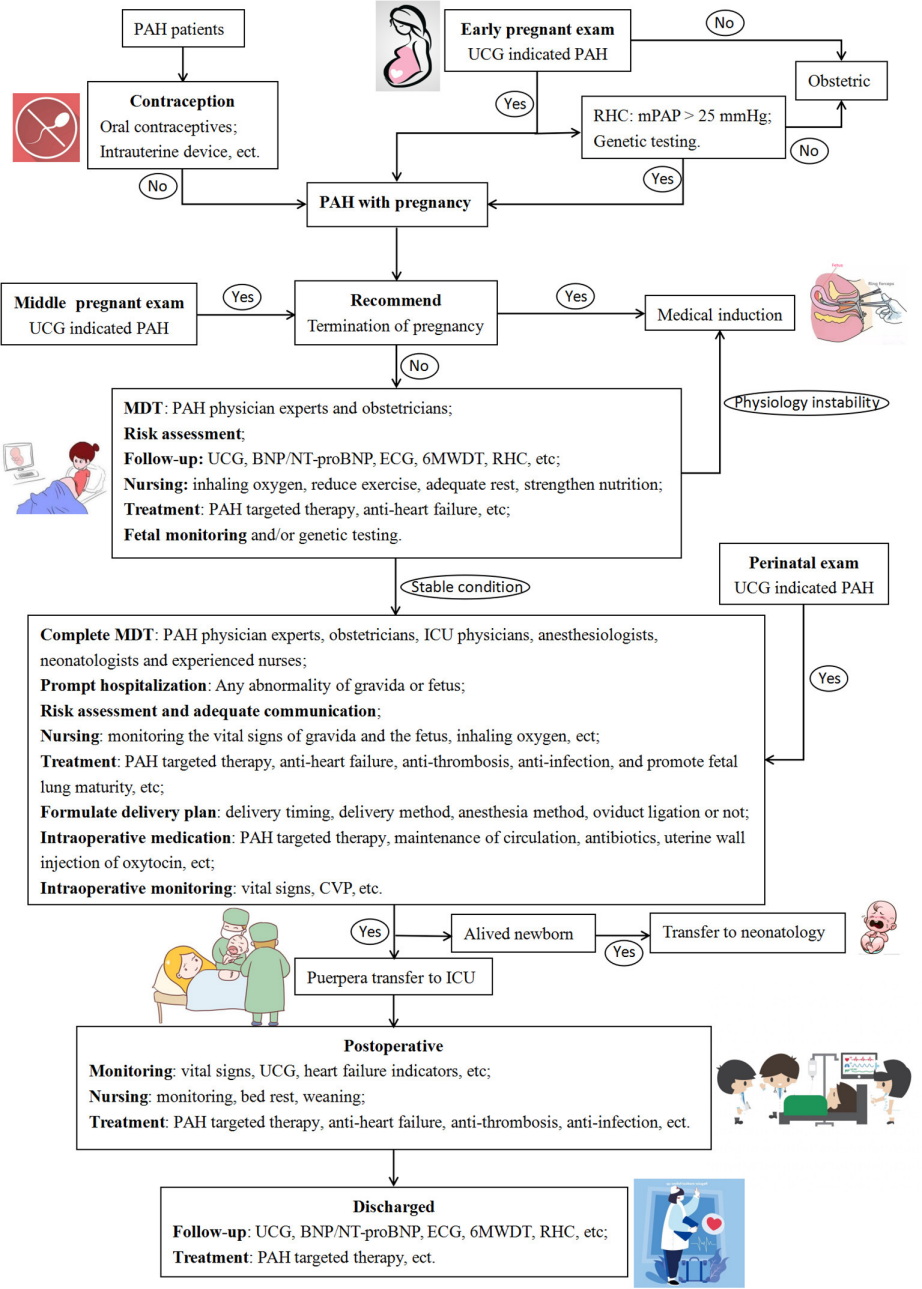
Flow chart of diagnosis and treatment for pregnant PAH patients. PAH, pulmonary arterial hypertension; UCG, ultrasound cardiogram; RHC, right heart catheter; mPAP, mean pulmonary artery pressure; MDT, multidisciplinary team; BNP, B-type natriuretic peptide; NT-proBNP, N terminal pro B-type natriuretic peptide; ECG, electrocardiogram; 6MWDT, six minutes walking distance test; ICU, intensive care unit; CVP, central venous pressure.

Complete MDT is essential for the comprehensive management of PAH patients during pregnancy (Figure 2) (24). All patients in the present study received tailor-made MDT management. Unfortunately, the start of monitoring time by the MDT team for pregnant women was relatively late in this center, which was detrimental to risk control during pregnancy. Because the maternal mortality rate remains high, patients with PAH are recommended to take contraception (7). Early maternity examinations for pregnant women are essential because PAH is more common in women, and the initial clinical manifestations may occur during pregnancy (7, 10, 11). For pregnant women, echocardiography is utilized to screen for PAH, which is non-invasive, repeatable, and easy to perform (7). With patient consent, it is recommended to perform invasive RHC at an experienced PAH center, and genetic testing can be performed if necessary (7). In this study, the SAE incidence of parturients with PAH was as high as 62.5%. Once PAH with pregnancy is diagnosed, patients need to be informed of the high risk of SAE during pregnancy and after delivery, and medical termination should be recommended. Patients who require continued pregnancy need to receive periodic follow-ups at PAH specialists and obstetricians, monitor PASP, right ventricular function, oxygen saturation, BNP/NT-proBNP, and fetal monitoring (7, 10, 11). During the follow-up, oxygen inhalation, adequate rest, and enhanced nutrition are required. Anti-PAH and anti-heart failure treatments are recommended to being actively given at least 3 months before delivery. Risk assessment should be performed monthly during pregnancy. Once the condition is unstable, emergency admission and prompt termination of the pregnancy are necessary.

No spontaneous abortion occurred in this study. Three patients chose medical induction at 11.3, 14.6, and 21.1 weeks. Two patients had intrauterine stillbirths during pregnancy, and cesarean section was performed at 31.6 and 24.9 weeks. Monitoring of pregnant women and fetuses during pregnancy is essential. The patients included in this study were all diagnosed with PAH and pregnancy when they were admitted to the hospital. PAH patients who aborted outside the hospital were not included. The abortion rate of women with PAH was not investigated in the present study. Complete MDT management is required during the third trimester and the entire perinatal period. Pregnant women with PAH should be hospitalized promptly when severe hypoxemia, deteriorated heart failure, coagulation dysfunction, hypertension, and fetal abnormalities occur. Communication should be strengthened with pregnant women and routine follow-up to help them fully understand their condition and improve the compliance of treatments. Pregnant women need to receive PAH-targeted therapy, anti-heart failure, anti-thrombosis, and anti-infection and to promote fetal lung maturity during the perinatal period. During normal pregnancy, blood flow and cardiac output will increase, reaching their peaks at approximately 32 and 24 weeks of gestation, respectively (25). Compared with normal pregnant women, patients with PAH-CHD are intolerant to this physiological change during pregnancy. The increase in PVR in pregnant women with PAH further aggravates PAH, overloads the right ventricle, and ultimately seriously affects right ventricular function (3). In the present study, the premature delivery rate of PAH patients who persisted in continuing pregnancy was high (66.7%). After discussion through MDT, they all chose to perform cesarean section with a mean gestational term of 32.37 weeks, and all of the preterm births were determined by MDT through discussion of the status of the pregnant woman and the fetus. The detailed delivery plan should be developed through MDT discussion, including delivery timing, delivery method, anesthesia method, and oviduct ligation; and communication with the patient to give a full sense of safety. Intraoperative monitoring of the patient's vital signs is important, and it is necessary to continue anti-PAH, maintain circulation, and anti-infective treatments (3). PAH patients are prone to hypoxemia (1), and this study found that it directly affects intraoperative bleeding. Intravenous administration of oxytocin may incr ease the burden on the heart of patients (26, 27), but the bleeding volume of 76.2% of patients in this study was well-controlled by injection of oxytocin through the uterine wall. However, there is currently no study on the dose of oxytocin related to uterine wall injection, and it needs to be administered reasonably according to the uterine contraction and the patient's condition. The highest-risk period for the patients is the puerperium and the early post-partum period (7). It is necessary to transfer to the ICU after delivery. PAH-targeted therapy, anti-heart failure, anti-thrombosis, and anti-infection therapies are recommended to be maintained until the patient is discharged. It is recommended that mothers with PAH avoid breastfeeding after delivery because pulmonary vasodilators may be excreted through breast milk (28). Breastfeeding can also increase maternal fatigue, which is detrimental to the post-partum recovery of PAH patients.

To improve the survival rate, it is recommended that neonates be transferred to neonatology after birth. Late hospitalization increases the risk of adverse events. Therefore, it is critical for patients with PAH in the middle and late stages of pregnancy to be surveillant and treated with the experienced MDT team.

### Limitations

The present study was a retrospective single-center trial and reported a relatively small number of patients, so recall bias or reporting bias are unlikely. Due to the small sample size, this study was unable to perform quantitative analysis and identify risk factors. Strengthening of vigilance and management of PAH patients with pregnancy are needed. The patients included in this study were mostly based on UCG and lacked hemodynamic parameters. Due to the small sample size, the failure to conduct a multifactor analysis of all indicators may cause statistical bias in the results. However, not only the baseline characteristics but also the vital signs during and after cesarean section in each patient were collected to investigate the potential risk factors for SAE.

## Conclusion

The survival rate of parturients and neonates with PAH has been improved due to MDT management and pulmonary vasodilator therapy during the perinatal period. However, PAH during pregnancy remains a substantial risk and commonly leads to SAE, especially in the early post-partum period. SpO_2_, ALB, PASP, BP, and coagulation function should be carefully monitored in pregnant PAH patients. Prospective and multicenter studies with large sample sizes in women with PAH are required to determine the pregnancy-related risk factors, supportive care strategies and advanced PAH therapy.

## Data Availability Statement

The original contributions presented in the study are included in the article/Supplementary Material, further inquiries can be directed to the corresponding authors.

## Ethics Statement

The studies involving human participants were reviewed and approved by the First Affiliated Hospital of Chongqing Medical University. Written informed consent for participation was not required for this study in accordance with the national legislation and the institutional requirements.

## Author Contributions

TS and PF were responsible for the study screening, data extraction, and writing the manuscript. XL contributed to data analysis. LW, HC, and YC were responsible for checking and reviewing the final manuscript. All authors have read and approved the final manuscript.

## Funding

This work was supported by the Chongqing Municipal Health and Health Committee (ZQNYXGDRCGZS2019001, Nos. 2019ZY3340 and 2016HBRC001).

## Conflict of Interest

The authors declare that the research was conducted in the absence of any commercial or financial relationships that could be construed as a potential conflict of interest.

## Publisher's Note

All claims expressed in this article are solely those of the authors and do not necessarily represent those of their affiliated organizations, or those of the publisher, the editors and the reviewers. Any product that may be evaluated in this article, or claim that may be made by its manufacturer, is not guaranteed or endorsed by the publisher.
