# Supplementary material for: Multidisciplinary Team Managements and Clinical Outcomes in Patients With Pulmonary Arterial Hypertension During the Perinatal Period
Source: Front Cardiovasc Med. 2021 Dec 17;8:795765. doi: 10.3389/fcvm.2021.795765 (PMC8718549; doi:10.3389/fcvm.2021.795765)
Supplement: Supplementary file 1 [file Table_1.DOCX]

**Supplementary materials online**

**Table S1.** Baseline characteristics in subgroups according to PAH etiology and gravidity history.

|  |  | PAH age, y | Pregnancy age, y | GW at hospitalization, wk | GW of delivery, wk | BMI, kg/m^2^ | HR, bpm | SBP, mmHg | DBP, mmHg |
| --- | --- | --- | --- | --- | --- | --- | --- | --- | --- |
| Total | N | 24 | 24 | 24 | 24 | 24 | 24 | 24 | 24 |
|  | Mean | 28.42 | 29.63 | 29.38 | 32.37 | 24.16 | 93.63 | 179.21 | 74.17 |
|  | SD | 6.00 | 4.87 | 8.63 | 7.20 | 4.76 | 12.40 | 293.01 | 15.26 |
| Aetiology |  |  |  |  |  |  |  |  |  |
| CHD | N | 19 | 19 | 19 | 19 | 19 | 19 | 19 | 19 |
|  | Mean | 28.05 | 29.47 | 30.26 | 33.34 | 24.15 | 96.32 | 197.68 | 74.84 |
|  | SD | 6.38 | 5.08 | 8.70 | 6.56 | 4.77 | 12.18 | 328.44 | 16.49 |
| CTD | N | 1 | 1 | 1 | 1 | 1 | 1 | 1 | 1 |
|  | Mean | 31 | 31 | 35.6 | 37 | 21.0938 | 79 | 92 | 68 |
|  | SD | . | . | . | . | . | . | . | . |
| IPAH | N | 4 | 4 | 4 | 4 | 4 | 4 | 4 | 4 |
|  | Mean | 29.50 | 30.00 | 23.63 | 26.60 | 24.93 | 84.50 | 113.25 | 72.50 |
|  | SD | 5.20 | 5.10 | 7.43 | 9.08 | 5.75 | 7.85 | 21.72 | 11.56 |
| Gravidity history |  |  |  |  |  |  |  |  |  |
| First | N | 9 | 9 | 9 | 9 | 9 | 9 | 9 | 9 |
|  | Mean | 27.22 | 27.78 | 28.06 | 29.10 | 22.41 | 93.00 | 278.22 | 76.44 |
|  | SD | 6.83 | 6.16 | 10.36 | 10.46 | 3.70 | 13.66 | 477.83 | 17.10 |
| Multiple | N | 15 | 15 | 15 | 15 | 15 | 15 | 15 | 15 |
|  | Mean | 29.13 | 30.73 | 30.17 | 34.33 | 25.20 | 94.00 | 119.80 | 72.80 |
|  | SD | 5.57 | 3.71 | 7.69 | 3.43 | 5.12 | 12.07 | 22.19 | 14.49 |

Abbreviation: PAH, pulmonary arterial hypertension; CHD, congenital heart disease; CTD, connective tissue disease; IPAH, idiopathic pulmonary arterial hypertension; N, number of pregnancies; SD, standard deviation; PAH, pulmonary arterial hypertension; y, years old; GW, gestational week; wk, week; BMI, body mass index; HR, heart rate; bpm, beats per minute; SBP, systolic blood pressure; DBP, diastolic pressure.

No statistical difference among all subgroups (P > 0.05).

**Table S2.** Clinical symptoms, electrocardiogram report, and RHC on admission of PAH patients with pregnancy.

| **Patients** | **Clinical symptoms** | **Electrocardiogram report** | **RHC** |
| --- | --- | --- | --- |
| **No.1** | Decreased exercise tolerance, short of breath, cyanosis, acropachy. | No obvious abnormality. | No |
| **No.2** | Decreased exercise tolerance, short of breath, cyanosis. | No obvious abnormality. | Yes |
| **No.3** | Short of breath, **thorcalgia.** | Sinus tachycardia. | No |
| **No.4** | Decreased exercise tolerance, short of breath. | No obvious abnormality. | Yes |
| **No.4*** | Decreased exercise tolerance, short of breath, palpitation. | No obvious abnormality. | Yes |
| **No.5** | Decreased exercise tolerance, short of breath. | No obvious abnormality. | No |
| **No.6** | Decreased exercise tolerance, short of breath. | Complete right bundle branch block. | Yes |
| **No.7** | Dyspnea in semireclining position, decreased exercise tolerance, short of breath, palpitation, acropachy. | Complete right bundle branch block. | No |
| **No.8** | Decreased exercise tolerance. | Sinus tachycardia. | No |
| **No.9** | Decreased exercise tolerance. | **Left anterior fascicular block.** | No |
| **No.10** | Dyspnea in semireclining position, decreased exercise tolerance, short of breath. | Sinus tachycardia. | No |
| **No.11** | Decreased exercise tolerance, short of breath. | Incomplete right bundle branch block. | No |
| **No.12** | Short of breath, palpitation. | Sinus tachycardia. | No |
| **No.13** | Short of breath, palpitation. | Sinus tachycardia, complete right bundle branch block. | No |
| **No.14** | Decreased exercise tolerance, short of breath, cyanosis. | No obvious abnormality. | Yes |
| **No.14*** | Decreased exercise tolerance, short of breath, syncope, hemoptysis, hoarseness, cyanosis, acropachy. | No obvious abnormality. | Yes |
| **No.15** | Decreased exercise tolerance, short of breath. | No obvious abnormality. | No |
| **No.16** | Decreased exercise tolerance, short of breath. | No obvious abnormality. | Yes |
| **No.17** | Decreased exercise tolerance, short of breath, palpitation, edema. | No obvious abnormality. | No |
| **No.18** | Dyspnea in semireclining position, decreased exercise tolerance, short of breath, edema. | Sinus tachycardia, l**eft anterior fascicular block,** complete right bundle branch block. | No |
| **No.19** | Dyspnea in semireclining position, decreased exercise tolerance, short of breath, hemoptysis. | No obvious abnormality. | No |
| **No.20** | Decreased exercise tolerance, short of breath. | Sinus tachycardia. | No |
| **No.21** | Asymptomatic. | No obvious abnormality. | No |
| **No.22** | Asymptomatic. | Sinus tachycardia. | No |

Abbreviation: PAH, pulmonary arterial hypertension; RHC, right-sided heart catheterization.

* The second pregnancy during the study period.

**Table S3.** Echocardiographic or RHC parameters at the time of diagnosis of PAH patients.

| **Patients** | **Echocardiographic** | | | | | | | |  | **RHC** | | | | | | | |
| --- | --- | --- | --- | --- | --- | --- | --- | --- | --- | --- | --- | --- | --- | --- | --- | --- | --- |
|  | **Test age,**  **y** | **PASP, mmHg** | **EF, %** | **LA diameter, mm** | **LV diameter, mm** | **RA diameter, mm** | **RV diameter, mm** | **PA diameter, mm** |  | **Test age,**  **y** | **mPAP,**  **mmHg** | **PASP,**  **mmHg** | **PCWP,**  **mmHg** | **RVSP,**  **mmHg** | **CO,**  **L/min** | **Qp,**  **L/min** | **Qs,**  **L/min** |
| **No.1** | 30 | 161.2 | 59 | 28 | 46 | 46 | 24 | 32 |  | - | - | - | - | - | - | - | - |
| **No.2** | 29 | 65 | 55 | 29 | 46 | 40 | 24 | 38 |  | 29 | 66 | 86 | - | - | - | 2.97 | 2.8 |
| **No.3** | 31 | 70.1 | 72 | 24 | 39 | 42 | 24 | - |  | - | - | - | - | - | - | - | - |
| **No.4** | 28 | 96.1 | 62 | 24 | 42 | 34 | 25 | 27 |  | 28 | 50 | 61 | 10 | 64 | 6 | - | - |
| **No.4*** | 29 | 79 | 68 | 29 | 43 | 42 | 28 | 43 |  | 28 | 50 | 61 | 10 | 64 | 6 | - | - |
| **No.5** | 40 | 71.5 | 67 | 42 | 42 | 30 | 27 | - |  | - | - | - | - | - | - | - | - |
| **No.6** | 30 | 55 | 72 | 25 | 40 | 34 | 18 | - |  | 31 | 47 | 57 | 8 | 47 | 5.6 | - | - |
| **No.7** | 30 | 71 | - | 43 | - | 45 | - | - |  | - | - | - | - | - | - | - | - |
| **No.8** | 25 | 113 | 56 | 34 | 50 | 48 | 25 | - |  | - | - | - | - | - | - | - | - |
| **No.9** | 36 | 61.4 | 66 | 26 | 52 | 42 | 23 | - |  | - |  |  |  |  |  | - |  |
| **No.10** | 31 | 63 | 36 | 45 | 66 | 34 | 19 | - |  | - | - | - | - | - | - | - | - |
| **No.11** | 30 | 41 | 56 | 32 | 44 | 48 | 38 | 25 |  | - | - | - | - | - | - | - | - |
| **No.12** | 20 | 44 | 56 | 27 | 44 | 52 | 43 | - |  | - | - | - | - | - | - | - | - |
| **No.13** | 22 | 50 | 54 | 32 | 45 | 50 | 28 | 36 |  | - | - | - | - | - | - | - | - |
| **No.14** | 22 | 61 | 72 | 24 | 41 | 30 | 17 | 25 |  | 23 | 71 | 78 | 9 | 64 | - | 4.19 | 4 |
| **No.14*** | 26 | 94.6 | 69 | 24 | 41 | 35 | 23 | 25 |  | 23 | 71 | 78 | 9 | 64 | - | 4.19 | 4 |
| **No.15** | 38 | 30 | 65 | 29 | 45 | 39 | 32 | - |  | - | - | - | - | - | - | - | - |
| **No.16** | 25 | 76.7 | 61 | 30 | 45 | 39 | 25 | - |  | 26 | 92 | 128 | 15 | 127 | - | 2.04 | 5.45 |
| **No.17** | 25 | 35 | 64 | 36 | 49 | 37 | 19 | - |  | - | - | - | - | - | - | - | - |
| **No.18** | 30 | 120 | 62 | 41 | 60 | 49 | 26 | 45 |  | - | - | - | - | - | - | - | - |
| **No.19** | 37 | 38 | 69 | 34 | 50 | 33 | 18 | - |  | - | - | - | - | - | - | - | - |
| **No.20** | 32 | 35 | 47 | 34 | 63 | 34 | 20 |  |  | - | - | - | - | - | - | - | - |
| **No.21** | 28 | 36 | 61 | 25 | 47 | 44 | 32 | - |  | - | - | - | - | - | - | - | - |
| **No.22** | 31 | 55 | 68 | 31 | 35 | 46 | 29 | 26 |  | - | - | - | - | - | - | - | - |

Abbreviation: RHC, right heart catheterization; PAH, pulmonary arterial hypertension; y, years old; PASP, pulmonary artery systolic pressure; EF, left ventricular ejection fraction; LA, left atrium; LV, left ventricle; RA, right atrium; RV, right ventricle; PA, main pulmonary artery; mPAP, mean pulmonary artery pressure; PCWP, pulmonary capillary wedge pressure; RVSP, right ventricular systolic pressure; CO, cardiac output; Qp, pulmonary blood flow; Qs, systemic blood flow.

**Table S4.** Summary of admission examination indicators for subgroups according to PAH etiology and gravidity history.

|  |  | SpO_2_, % | HB, g/L | PLT, ×10^9^/L | PT, s | APTT, s | Fbg, g/L | ALB, g/L | Braden score | Barthel index | PASP, mmHg | EF, % | LA diameter, mm | LV diameter, mm | RA diameter, mm | RV diameter, mm | PA diameter, mm |
| --- | --- | --- | --- | --- | --- | --- | --- | --- | --- | --- | --- | --- | --- | --- | --- | --- | --- |
| Total | N | 24 | 24 | 24 | 24 | 24 | 24 | 24 | 24 | 24 | 24 | 23 | 24 | 23 | 24 | 23 | 19 |
|  | Mean | 92.54 | 119.33 | 217.25 | 11.68 | 29.57 | 3.99 | 33.79 | 14.88 | 90.00 | 73.74 | 63.22 | 32.29 | 46.70 | 41.21 | 26.70 | 30.42 |
|  | SD | 9.40 | 33.03 | 154.77 | 1.63 | 5.89 | 0.79 | 5.42 | 2.33 | 15.11 | 27.35 | 6.30 | 7.03 | 8.32 | 9.34 | 7.06 | 6.45 |
| Aetiology |  |  |  |  |  |  |  |  |  |  |  |  |  |  |  |  |  |
| CHD | N | 19 | 19 | 19 | 19 | 19 | 19 | 19 | 19 | 19* | 19 | 18 | 19 | 18 | 19 | 18 | 15 |
|  | Mean | 91.74 | 125.00 | 183.05 | 11.61 | 30.16 | 3.88 | 34.79 | 14.47 | 93.42 | 72.46 | 62.22 | 33.00 | 46.89 | 41.79 | 27.28 | 30.07 |
|  | SD | 10.14 | 29.13 | 58.14 | 1.62 | 6.19 | 0.80 | 5.01 | 2.12 | 6.02 | 29.01 | 6.69 | 7.44 | 8.80 | 10.27 | 7.74 | 6.03 |
| CTD | N | 1 | 1 | 1 | 1 | 1 | 1 | 1 | 1 | 1* | 1 | 1 | 1 | 1 | 1 | 1 | 1 |
|  | Mean | 99 | 126 | 212 | 10.9 | 28 | 4.96 | 30 | 18 | 30 | 103 | 69 | 27 | 39 | 45 | 28 | 34 |
|  | SD | . | . | . | . | . | . | . | . | . | . | . | . | . | . | . | . |
| IPAH | N | 4 | 4 | 4 | 4 | 4 | 4 | 4 | 4 | 4* | 4 | 4 | 4 | 4 | 4 | 4 | 3 |
|  | Mean | 94.75 | 90.75 | 381.00 | 12.23 | 27.15 | 4.29 | 30.00 | 16.00 | 88.75 | 72.48 | 66.25 | 30.25 | 47.75 | 37.50 | 23.75 | 31.00 |
|  | SD | 5.97 | 44.36 | 346.71 | 2.01 | 5.02 | 0.65 | 6.68 | 2.94 | 16.01 | 19.63 | 2.87 | 5.38 | 6.90 | 3.42 | 3.50 | 10.58 |
| Gravidity history | |  |  |  |  |  |  |  |  |  |  |  |  |  |  |  |  |
| First | N | 9 | 9 | 9 | 9 | 9 | 9 | 9 | 9 | 9 | 9 | 62.33 | 9 | 43 | 9 | 27.67 | 28.14 |
|  | Mean | 96.78 | 120.33 | 185.11 | 11.71 | 28.92 | 3.92 | 36.78 | 14.44 | 92.78 | 70.76 | 9.00 | 31.44 | 9.00 | 38.11 | 9.00 | 7.00 |
|  | SD | 2.68 | 21.31 | 43.11 | 1.05 | 5.16 | 0.75 | 3.38 | 1.88 | 7.95 | 24.92 | 6.36 | 6.17 | 7.45 | 10.33 | 9.30 | 4.02 |
| Multiple | N | 15 | 15 | 15 | 15 | 15 | 15 | 15 | 15 | 15 | 15 | 63.79 | 15 | 49.07 | 15 | 26.07 | 31.75 |
|  | Mean | 90 | 118.73 | 236.53 | 11.67 | 29.95 | 4.03 | 32.00 | 15.13 | 88.33 | 75.53 | 14.00 | 32.80 | 14.00 | 43.07 | 14.00 | 12.00 |
|  | SD | 11.07 | 39.14 | 192.95 | 1.94 | 6.43 | 0.84 | 5.71 | 2.59 | 18.19 | 29.40 | 6.44 | 7.66 | 8.21 | 8.52 | 5.48 | 7.35 |

Abbreviation: PAH, pulmonary arterial hypertension; CHD, congenital heart disease; CTD, connective tissue disease; IPAH, idiopathic pulmonary arterial hypertension; N, number of pregnancies; SD, standard deviation; SpO_2_, percutaneous oxygen saturation; HB, hemoglobin; PLT, platelet; PT, coagulation time; APTT, Partial clotting time; Fbg, fibrinogen; ALB, albumin; PASP, pulmonary artery systolic pressure; EF, left ventricular ejection fraction; LA, left atrium; LV, left ventricle; RA, right atrium; RV, right ventricle; PA, main pulmonary artery.

*The difference in barthel index among subgroups grouped by PAH aetiology was statistically significant (P < 0.05). No statistical difference in the other indicators among the subgroups (P > 0.05).

**Table S5.** Differences in admission examination indicators of different maternal outcomes.

|  |  | SpO_2_, % | HB, g/L | PLT, ×10^9^/L | PT, s | APTT, s | Fbg, g/L | ALB, g/L | Braden score | Barthel index | PASP, mmHg | EF, % | LA diameter, mm | LV diameter, mm | RA diameter, mm | RV diameter, mm | PA diameter, mm |
| --- | --- | --- | --- | --- | --- | --- | --- | --- | --- | --- | --- | --- | --- | --- | --- | --- | --- |
| SAE |  |  |  |  |  |  |  |  |  |  |  |  |  |  |  |  |  |
| No | N | 9 | 9 | 9 | 9 | 9 | 9 | 9 | 9 | 9 | 9 | 9 | 9 | 9 | 9 | 9* | 8 |
|  | Mean | 96.22 | 117.89 | 192.67 | 11.03 | 28.19 | 4.22 | 35.33 | 13.78 | 96.67 | 64.28 | 62.78 | 30.78 | 45.67 | 40.44 | 30.56 | 29.25 |
|  | SD | 5.45 | 16.71 | 52.86 | 0.77 | 3.23 | 0.47 | 2.40 | 1.09 | 3.54 | 18.48 | 7.26 | 5.85 | 9.43 | 6.02 | 7.57 | 6.76 |
| Yes | N | 15 | 15 | 15 | 15 | 15 | 15 | 15 | 15 | 15 | 15 | 14 | 15 | 14 | 15 | 14* | 11 |
|  | Mean | 90.33 | 120.20 | 232.00 | 12.07 | 30.39 | 3.85 | 32.87 | 15.53 | 86.00 | 79.41 | 63.5 | 33.20 | 47.36 | 41.67 | 24.21 | 31.27 |
|  | SD | 10.69 | 40.38 | 192.71 | 1.90 | 7.01 | 0.92 | 6.52 | 2.64 | 17.95 | 30.68 | 5.88 | 7.70 | 7.82 | 11.05 | 5.67 | 6.41 |
| Death |  |  |  |  |  |  |  |  |  |  |  |  |  |  |  |  |  |
| Alive | N | 22* | 22 | 22 | 22 | 22 | 22 | 22* | 22 | 22 | 22* | 22 | 22 | 22 | 22 | 22 | 17 |
|  | Mean | 94.86 | 115.86 | 225.55 | 11.68 | 28.95 | 4.07 | 34.45 | 14.95 | 90.00 | 69.89 | 63.36 | 31.82 | 46.55 | 40.68 | 26.91 | 30.59 |
|  | SD | 5.35 | 30.71 | 157.90 | 1.70 | 5.73 | 0.75 | 5.14 | 2.40 | 15.81 | 24.13 | 6.41 | 6.22 | 8.48 | 9.52 | 7.15 | 6.78 |
| Deceased | N | 2* | 2 | 2 | 2 | 2 | 2 | 2* | 2 | 2 | 2* | 1 | 2 | 1 | 2 | 1 | 2 |
|  | Mean | 67 | 157.50 | 126.00 | 11.75 | 36.40 | 3.08 | 26.50 | 14.00 | 90.00 | 116.05 | 60 | 37.50 | 50 | 47.00 | 22 | 29 |
|  | SD | 2.83 | 45.96 | 96.17 | 0.92 | 2.69 | 0.84 | 2.12 | 1.41 | 0.00 | 32.60 | . | 16.26 | . | 5.66 | . | 2.83 |
| HF |  |  |  |  |  |  |  |  |  |  |  |  |  |  |  |  |  |
| No | N | 13 | 13 | 13 | 13 | 13* | 13* | 13 | 13 | 13 | 13 | 13 | 13 | 13 | 13 | 13 | 11 |
|  | Mean | 95.38 | 113.46 | 248.85 | 11.20 | 27.38 | 4.39 | 34.54 | 15.46 | 88.46 | 70.43 | 63.62 | 30.77 | 45.85 | 39.62 | 28.23 | 28.73 |
|  | SD | 5.56 | 30.59 | 200.41 | 1.34 | 3.44 | 0.49 | 5.03 | 2.88 | 19.83 | 24.83 | 6.46 | 5.70 | 8.66 | 6.32 | 7.46 | 6.29 |
| Yes | N | 11 | 11 | 11 | 11 | 11* | 11* | 11 | 11 | 11 | 11 | 10 | 11 | 10 | 11 | 10 | 8 |
|  | Mean | 89.18 | 126.27 | 179.91 | 12.26 | 32.16 | 3.52 | 32.91 | 14.18 | 91.82 | 77.65 | 62.7 | 34.09 | 47.8 | 43.09 | 24.7 | 32.75 |
|  | SD | 11.97 | 35.91 | 63.78 | 1.82 | 7.22 | 0.84 | 5.97 | 1.25 | 6.81 | 30.81 | 6.40 | 8.25 | 8.18 | 12.07 | 6.31 | 6.30 |
| RF |  |  |  |  |  |  |  |  |  |  |  |  |  |  |  |  |  |
| No | N | 18* | 18 | 18 | 18 | 18* | 18 | 18 | 18 | 18* | 18* | 18 | 18 | 18 | 18* | 18 | 13 |
|  | Mean | 95.78 | 117.06 | 237.33 | 11.48 | 27.63 | 4.17 | 34.61 | 14.72 | 93.06 | 62.59 | 63.39 | 31.17 | 46.44 | 38.67 | 26.89 | 29.54 |
|  | SD | 4.78 | 32.03 | 172.67 | 1.27 | 4.46 | 0.63 | 4.47 | 2.42 | 9.26 | 15.25 | 6.73 | 5.93 | 8.58 | 8.19 | 7.87 | 6.25 |
| Yes | N | 6* | 6 | 6 | 6 | 6* | 6 | 6 | 6 | 6* | 6* | 5 | 6 | 5 | 6* | 5 | 6 |
|  | Mean | 82.83 | 126.17 | 157.00 | 12.28 | 35.37 | 3.47 | 31.33 | 15.33 | 80.83 | 107.18 | 62.6 | 35.67 | 47.6 | 48.83 | 26 | 30.42 |
|  | SD | 13.33 | 38.18 | 54.94 | 2.49 | 6.18 | 1.04 | 7.58 | 2.16 | 24.98 | 29.32 | 5.03 | 9.46 | 8.14 | 8.98 | 3.16 | 6.45 |
| Infection |  |  |  |  |  |  |  |  |  |  |  |  |  |  |  |  |  |
| No | N | 14* | 14 | 14 | 14* | 14* | 14 | 14 | 14 | 14 | 14* | 14 | 14 | 14 | 14 | 14 | 10 |
|  | Mean | 96.43 | 116.29 | 204.50 | 11.20 | 27.29 | 4.17 | 34.71 | 14.14 | 93.93 | 62.39 | 62.29 | 31.79 | 45.86 | 39.86 | 28.79 | 29.9 |
|  | SD | 4.38 | 16.98 | 52.17 | 0.72 | 3.07 | 0.56 | 3.17 | 1.29 | 6.56 | 16.30 | 7.09 | 5.93 | 9.20 | 8.30 | 7.82 | 6.23 |
| Yes | N | 10* | 10 | 10 | 10* | 10* | 10 | 10 | 10 | 10 | 10* | 9 | 10 | 9 | 10 | 9 | 9 |
|  | Mean | 87.10 | 123.60 | 235.10 | 12.36 | 32.76 | 3.74 | 32.50 | 15.90 | 84.50 | 89.62 | 64.67 | 33.00 | 48 | 43.10 | 23.44 | 31 |
|  | SD | 11.90 | 48.35 | 238.07 | 2.28 | 7.46 | 1.02 | 7.58 | 3.07 | 21.53 | 32.35 | 4.87 | 8.64 | 7.05 | 10.81 | 4.28 | 7.02 |

Abbreviation: SAE, serious adverse event; HF, heart failure; RF, respiratory failure; SD, standard deviation; N, number of pregnancies; SD, standard deviation; SpO_2_, percutaneous oxygen saturation; HB, hemoglobin; PLT, platelet; PT, coagulation time; APTT, Partial clotting time; Fbg, fibrinogen; ALB, albumin; PASP, pulmonary artery systolic pressure; EF, left ventricular ejection fraction; LA, left atrium; LV, left ventricle; RA, right atrium; RV, right ventricle; PA, main pulmonary artery.

* The difference between subgroups was statistically significant (P < 0.05).

**Table S6.** Differences in baseline characteristics and vital signs at the moment of fetal retrieval of different pregnancy maternal outcomes.

|  |  | Aetiology | Gravidity history | Arrhythmia | Delivery mode | Anesthesia | Admission | | |  | The moment of fetus retrieval | | | |
| --- | --- | --- | --- | --- | --- | --- | --- | --- | --- | --- | --- | --- | --- | --- |
|  |  |  |  |  |  |  | HR, bpm | SBP, mmHg | DBP, mmHg |  | SpO_2_, % | SBP, mmHg | DBP, mmHg | HR, bpm |
| SAE |  |  |  |  |  |  |  |  |  |  |  |  |  |  |
| No | N | 9 | 9 | 9 | 9 | 9 | 9 | 9 | 9 |  | 8 | 8 | 8 | 8 |
|  | Mean | 0.44 | 0.67 | 0.44 | 0.89 | 1.11 | 91.89 | 112.44 | 66.44 |  | 98.63 | 125.50 | 69.25 | 98.25 |
|  | SD | 0.88 | 0.50 | 0.53 | 0.33 | 0.78 | 10.27 | 13.32 | 8.90 |  | 2.72 | 21.89 | 8.60 | 15.33 |
| Yes | N | 15 | 15 | 15 | 15 | 15 | 15 | 15 | 15 |  | 13 | 13 | 13 | 13 |
|  | Mean | 0.33 | 0.60 | 0.53 | 0.87 | 1.73 | 94.67 | 126.20 | 78.80 |  | 97.15 | 130.46 | 73.15 | 91.08 |
|  | SD | 0.72 | 0.51 | 0.52 | 0.35 | 1.10 | 13.76 | 26.37 | 16.61 |  | 5.41 | 26.38 | 13.79 | 17.08 |
| Death |  |  |  |  |  |  |  |  |  |  |  |  |  |  |
| Alive | N | 22 | 22 | 22 | 22 | 22* | 22 | 22* | 22* |  | 19* | 19 | 19 | 19 |
|  | Mean | 0.41 | 0.59 | 0.50 | 0.86 | 1.36 | 93.32 | 117.64 | 72.09 |  | 98.84 | 127.37 | 70.74 | 94.42 |
|  | SD | 0.80 | 0.50 | 0.51 | 0.35 | 0.95 | 12.90 | 19.69 | 13.59 |  | 2.22 | 24.24 | 12.00 | 17.19 |
| Deceased | N | 2 | 2 | 2 | 2 | 2* | 2 | 2* | 2* |  | 2* | 2 | 2 | 2 |
|  | Mean | 0.00 | 1.00 | 0.50 | 1.00 | 3.00 | 97.00 | 158.50 | 97.00 |  | 87.00 | 140.00 | 80.50 | 88.00 |
|  | SD | 0.00 | 0.00 | 0.71 | 0.00 | 0.00 | 4.24 | 31.82 | 18.39 |  | 8.49 | 31.11 | 10.61 | 2.83 |
| HF |  |  |  |  |  |  |  |  |  |  |  |  |  |  |
| No | N | 13 | 13 | 13 | 13 | 13 | 13 | 13* | 13* |  | 12 | 12* | 12* | 12 |
|  | Mean | 0.54 | 0.69 | 0.54 | 0.92 | 1.46 | 90.85 | 111.38 | 68.31 |  | 98.58 | 118.92 | 66.50 | 92.92 |
|  | SD | 0.88 | 0.48 | 0.52 | 0.28 | 0.97 | 10.18 | 12.71 | 8.89 |  | 2.68 | 22.51 | 9.31 | 15.25 |
| Yes | N | 11 | 11 | 11 | 11 | 11 | 11 | 11* | 11* |  | 9 | 9* | 9* | 9 |
|  | Mean | 0.18 | 0.55 | 0.45 | 0.82 | 1.55 | 96.91 | 132.45 | 81.09 |  | 96.56 | 141.44 | 78.56 | 95.00 |
|  | SD | 0.60 | 0.52 | 0.52 | 0.41 | 1.13 | 14.41 | 27.63 | 18.52 |  | 6.27 | 21.35 | 12.12 | 18.78 |
| RF |  |  |  |  |  |  |  |  |  |  |  |  |  |  |
| No | N | 18 | 18 | 18 | 18 | 18 | 18 | 18 | 18 |  | 15* | 15 | 15 | 15 |
|  | Mean | 0.44 | 0.56 | 0.39 | 0.83 | 1.28 | 92.06 | 119.72 | 72.61 |  | 98.93 | 128.80 | 71.33 | 93.60 |
|  | SD | 0.86 | 0.51 | 0.50 | 0.38 | 0.96 | 11.86 | 20.28 | 14.30 |  | 2.09 | 21.12 | 9.94 | 15.45 |
| Yes | N | 6 | 6 | 6 | 6 | 6 | 6 | 6 | 6 |  | 6* | 6 | 6 | 6 |
|  | Mean | 0.17 | 0.83 | 0.83 | 1.00 | 2.17 | 98.33 | 125.00 | 78.83 |  | 94.67 | 128.00 | 72.50 | 94.33 |
|  | SD | 0.41 | 0.41 | 0.41 | 0.00 | 0.98 | 13.92 | 31.94 | 18.45 |  | 7.42 | 33.41 | 17.24 | 20.29 |
| Infection |  |  |  |  |  |  |  |  |  |  |  |  |  |  |
| No | N | 14 | 14 | 14 | 14 | 14* | 14 | 14 | 14 |  | 12 | 12 | 12 | 12 |
|  | Mean | 0.43 | 0.50 | 0.43 | 0.86 | 1.14 | 95.21 | 122.43 | 74.71 |  | 98.75 | 130.67 | 71.92 | 97.67 |
|  | SD | 0.85 | 0.52 | 0.51 | 0.36 | 0.77 | 10.33 | 22.33 | 14.80 |  | 2.30 | 23.22 | 11.07 | 14.35 |
| Yes | N | 10 | 10 | 10 | 10 | 10* | 10 | 10 | 10 |  | 9 | 9 | 9 | 9 |
|  | Mean | 0.30 | 0.80 | 0.60 | 0.90 | 2.00 | 91.40 | 119.10 | 73.40 |  | 96.33 | 125.78 | 71.33 | 88.67 |
|  | SD | 0.68 | 0.42 | 0.52 | 0.32 | 1.16 | 15.15 | 25.10 | 16.66 |  | 6.38 | 26.86 | 13.80 | 18.43 |

Abbreviation: SAE, serious adverse event; HF, heart failure; RF, respiratory failure; N, number of pregnancies; SD, standard deviation; HR, heart rate; bpm, beat per minute; SBP, systolic blood pressure; DBP, diastolic pressure.

* The difference between subgroups was statistically significant (P < 0.05).

**Figure S1-S21.** Intraoperative management of cesarean section for PAH patients pregnant under anesthesia.
