# Supplementary material for: Multidisciplinary Team Managements and Clinical Outcomes in Patients With Pulmonary Arterial Hypertension During the Perinatal Period
Source: Front Cardiovasc Med. 2021 Dec 17;8:795765. doi: 10.3389/fcvm.2021.795765 (PMC8718549; doi:10.3389/fcvm.2021.795765)
Supplement: Supplementary file 2 [file Data_Sheet_1.PDF]

Patient: No.1

Anesthesia: TGA

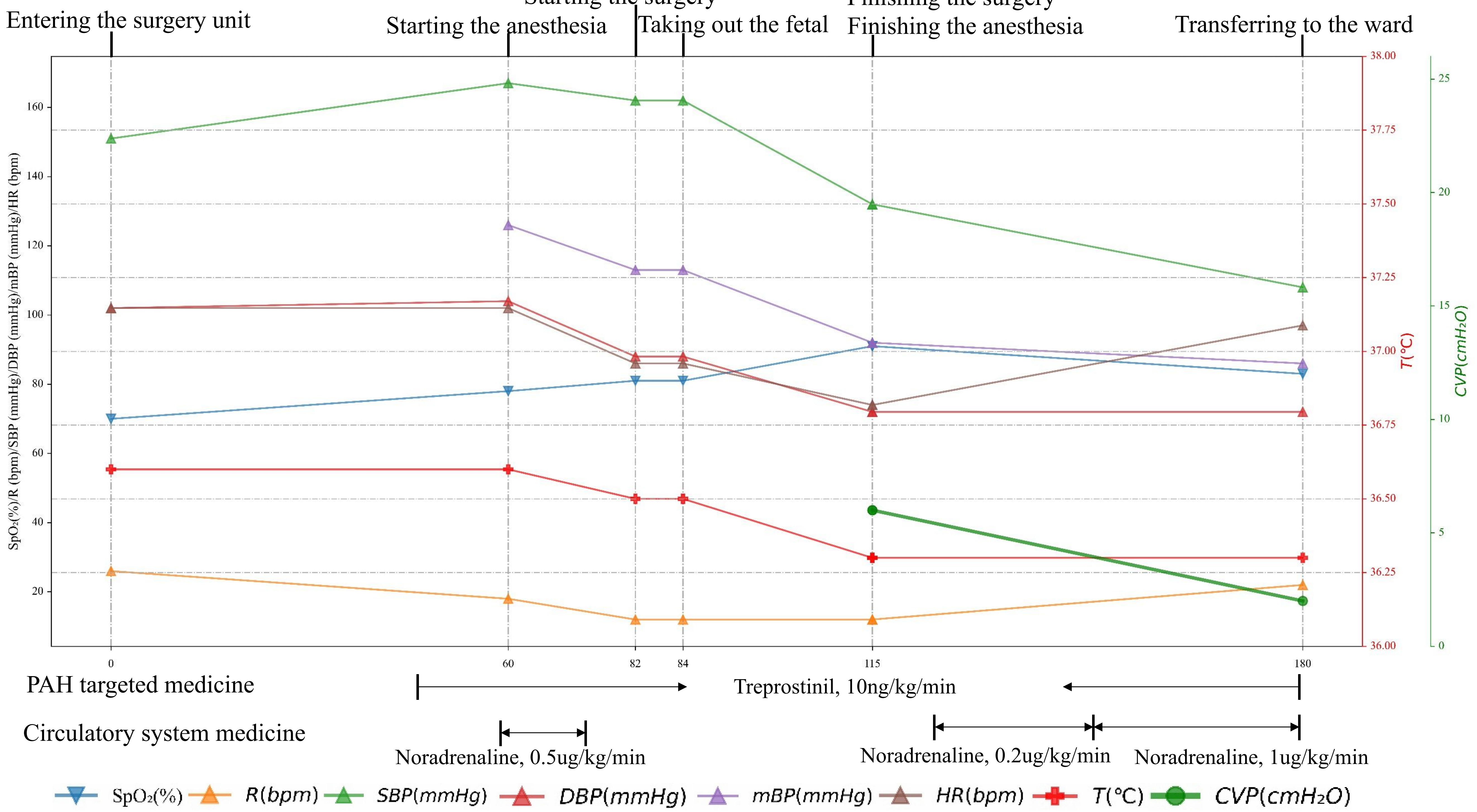

Figure S1

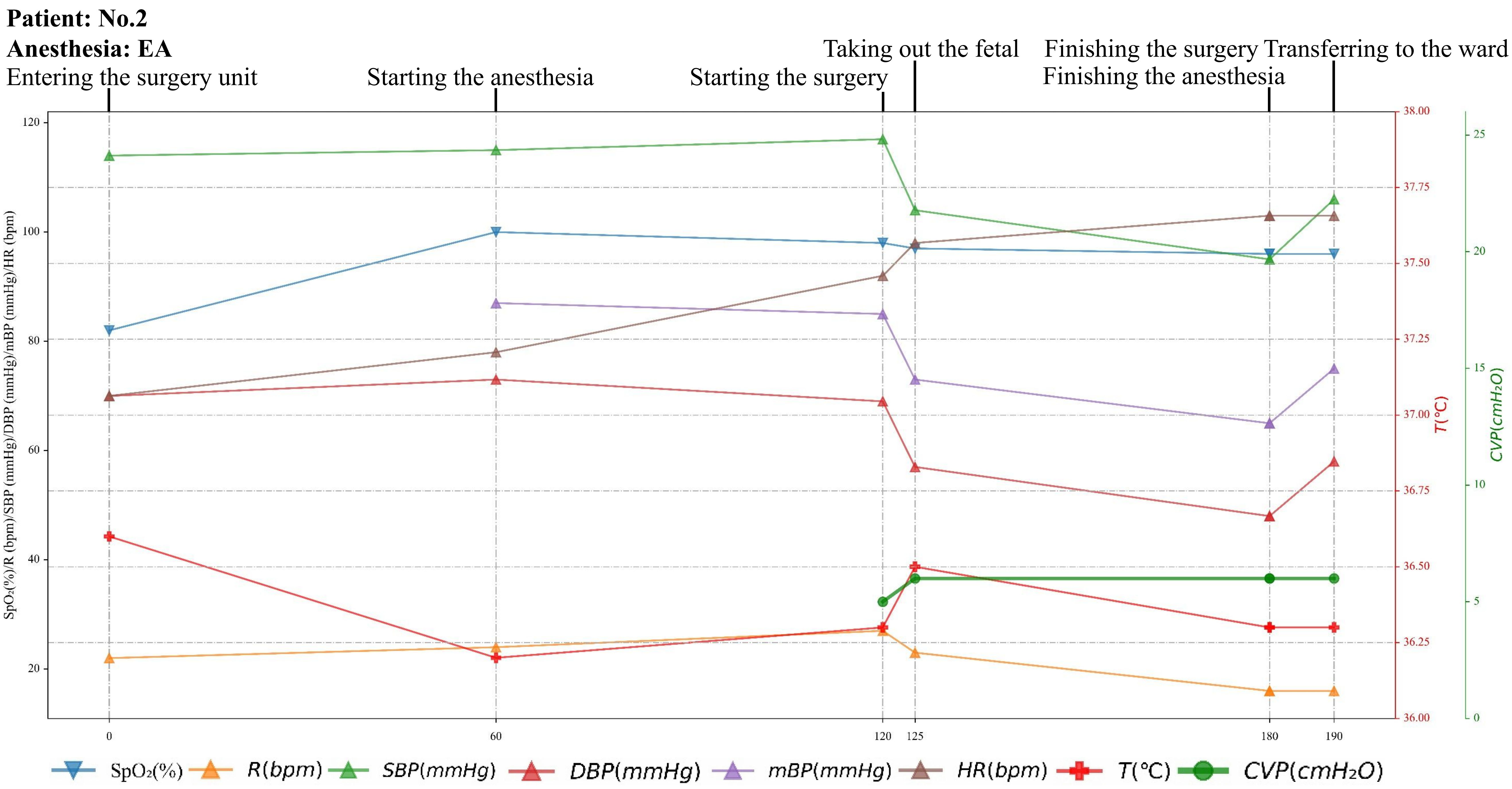
Patient: No.2
Anesthesia: EA
Entering the surgery unit
Starting the anesthesia
Starting the surgery
Taking out the fetal
Finishing the surgery
Finishing the anesthesia
Transferring to the ward
SpO2(%)/R (bpm)/SBP (mmHg)/DBP (mmHg)/mBP (mmHg)/HR (bpm)
T(°C)
CVP(cmH2O)
SpO2(%)
R(bpm)
SBP(mmHg)
DBP(mmHg)
mBP(mmHg)
HR(bpm)
T(°C)
CVP(cmH2O)

Figure S2

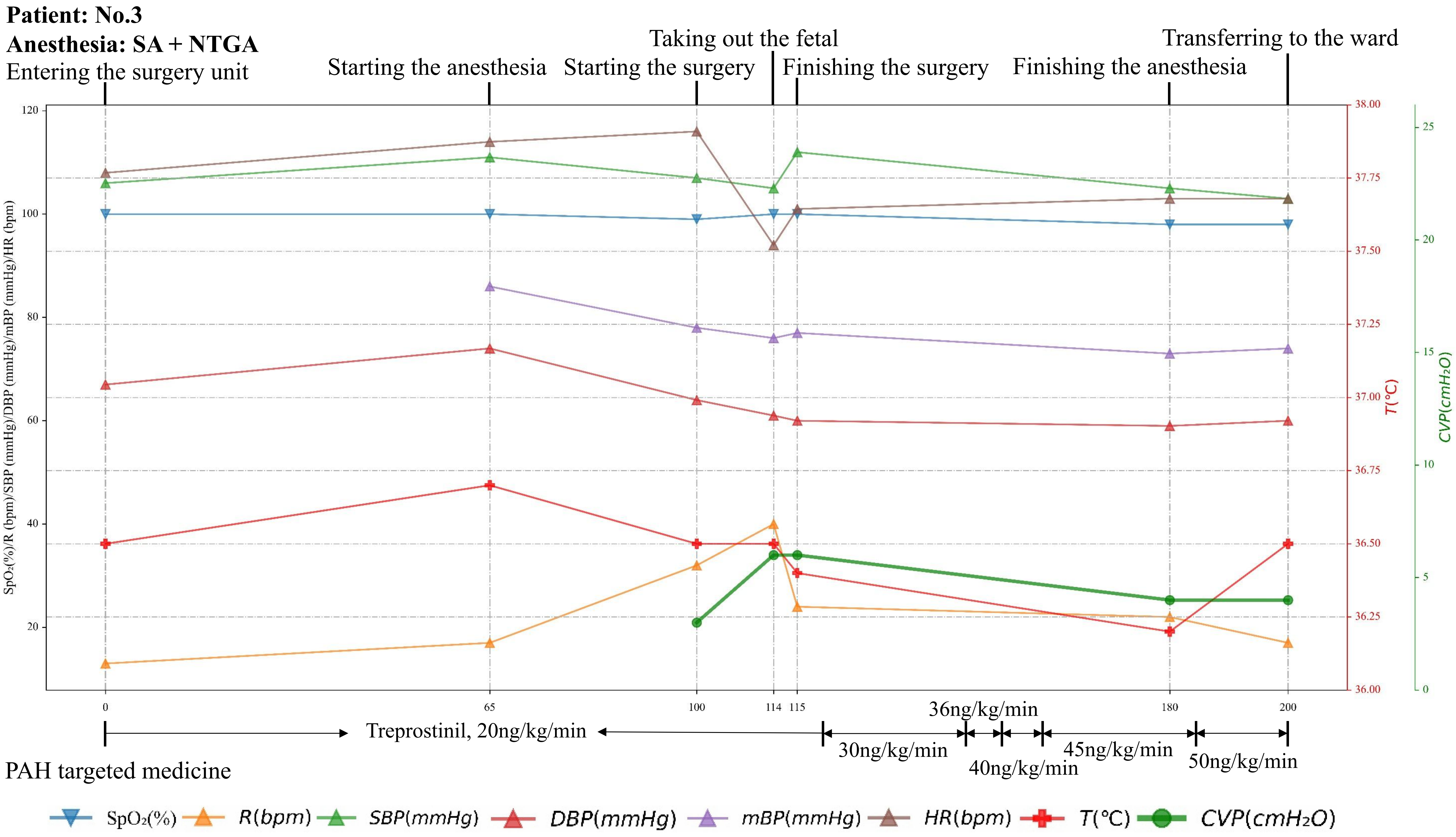

Patient: No.3
Anesthesia: SA + NTGA
Entering the surgery unit
Starting the anesthesia
Starting the surgery
Taking out the fetal
Finishing the surgery
Finishing the anesthesia
Transferring to the ward
SpO₂(%)/R (bpm)/SBP (mmHg)/DBP (mmHg)/mBP (mmHg)/HR (bpm)
T(°C)
CVP(cmH₂O)
0
65
100
114
115
180
200
Treprostinil, 20ng/kg/min
30ng/kg/min
36ng/kg/min
40ng/kg/min
45ng/kg/min
50ng/kg/min
PAH targeted medicine
SpO₂(%)
R(bpm)
SBP(mmHg)
DBP(mmHg)
mBP(mmHg)
HR(bpm)
T(°C)
CVP(cmH₂O)

Figure S3

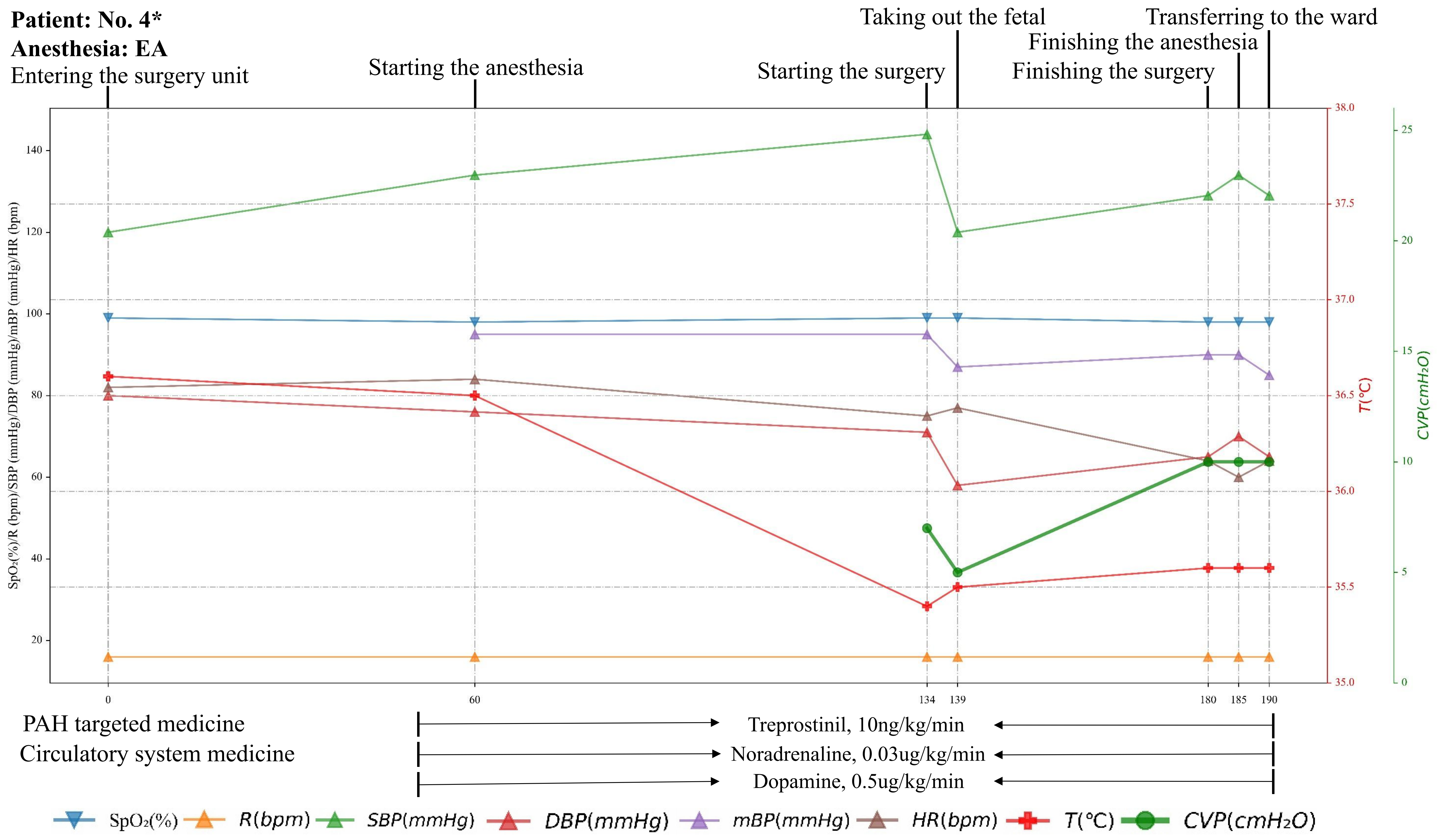
Patient: No. 4*
Anesthesia: EA
Entering the surgery unit
Starting the anesthesia
Starting the surgery
Taking out the fetal
Finishing the surgery
Finishing the anesthesia
Transferring to the ward
SpO₂(%)/R (bpm)/SBP (mmHg)/DBP (mmHg)/mBP (mmHg)/HR (bpm)
T(°C)
CVP(cmH₂O)
0
60
134
139
180
185
190
PAH targeted medicine
Circulatory system medicine
Treprostinil, 10ng/kg/min
Noradrenaline, 0.03ug/kg/min
Dopamine, 0.5ug/kg/min
SpO₂(%)
R(bpm)
SBP(mmHg)
DBP(mmHg)
mBP(mmHg)
HR(bpm)
T(°C)
CVP(cmH₂O)

Figure S4

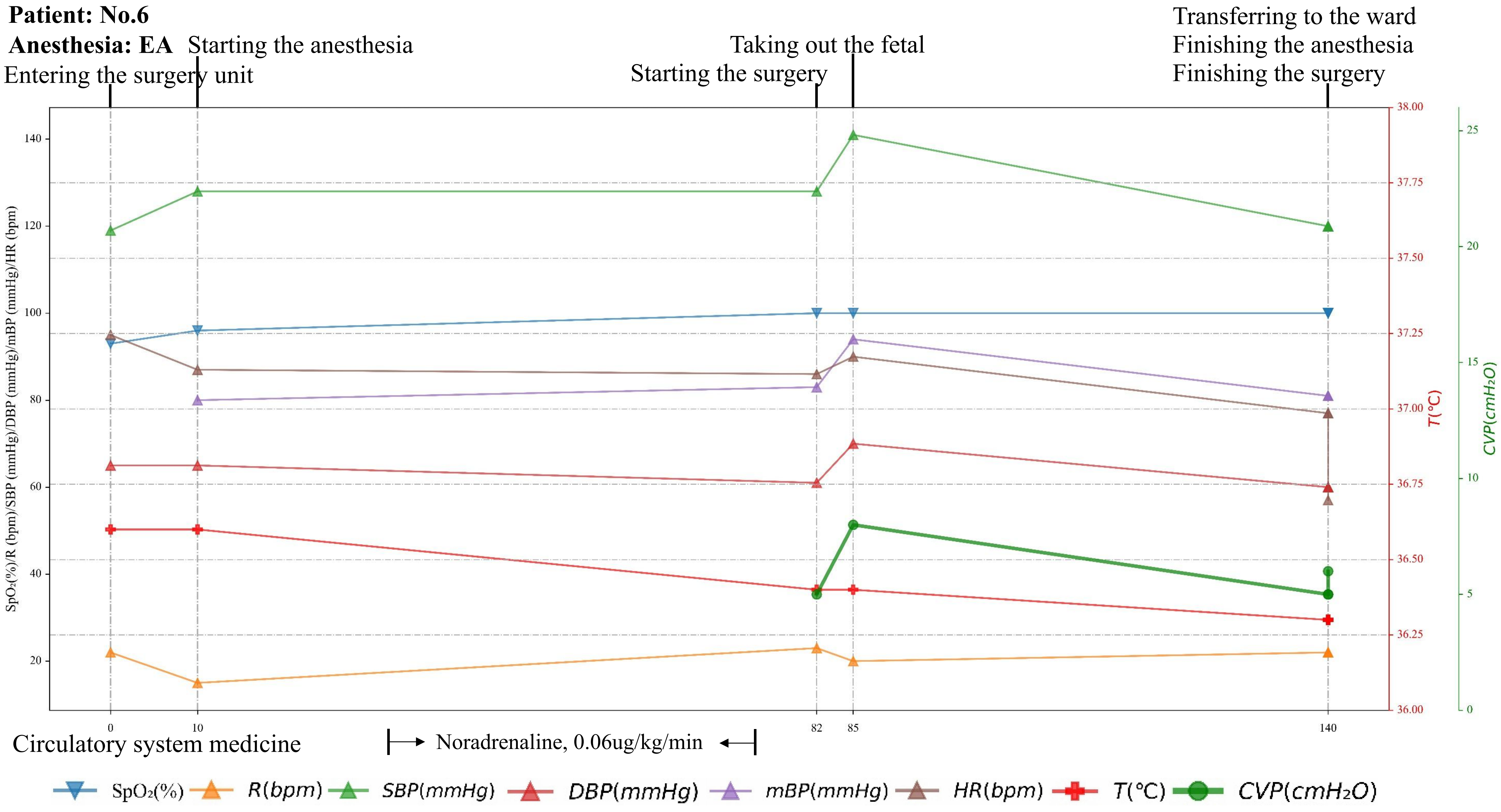
Patient: No.6
Anesthesia: EA
Starting the anesthesia
Entering the surgery unit
Taking out the fetal
Starting the surgery
Transferring to the ward
Finishing the anesthesia
Finishing the surgery
SpO₂(%)/R (bpm)/SBP (mmHg)/DBP (mmHg)/mBP (mmHg)/HR (bpm)
T(°C)
CVP(cmH₂O)
Circulatory system medicine
Noradrenaline, 0.06ug/kg/min
SpO₂(%)
R(bpm)
SBP(mmHg)
DBP(mmHg)
mBP(mmHg)
HR(bpm)
T(°C)
CVP(cmH₂O)

Figure S5

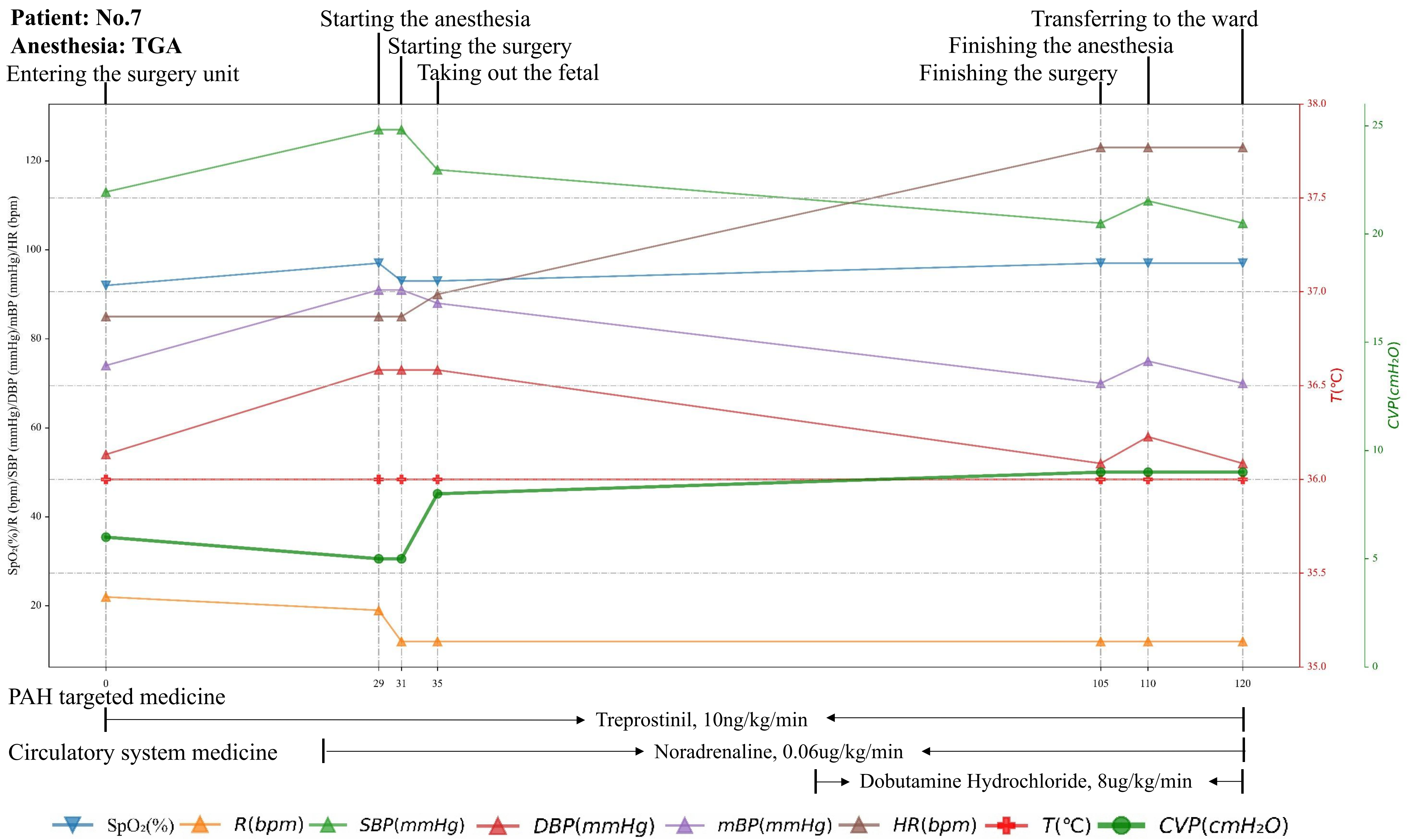

Patient: No.7
Anesthesia: TGA
Entering the surgery unit
Starting the anesthesia
Starting the surgery
Taking out the fetal
Finishing the surgery
Finishing the anesthesia
Transferring to the ward
SpO2(%)/R (bpm)/SBP (mmHg)/DBP (mmHg)/mBP (mmHg)/HR (bpm)
T(°C)
CVP(cmH2O)
PAH targeted medicine
Treprostinil, 10ng/kg/min
Circulatory system medicine
Noradrenaline, 0.06ug/kg/min
Dobutamine Hydrochloride, 8ug/kg/min
SpO2(%) R(bpm) SBP(mmHg) DBP(mmHg) mBP(mmHg) HR(bpm) T(°C) CVP(cmH2O)

Figure S6

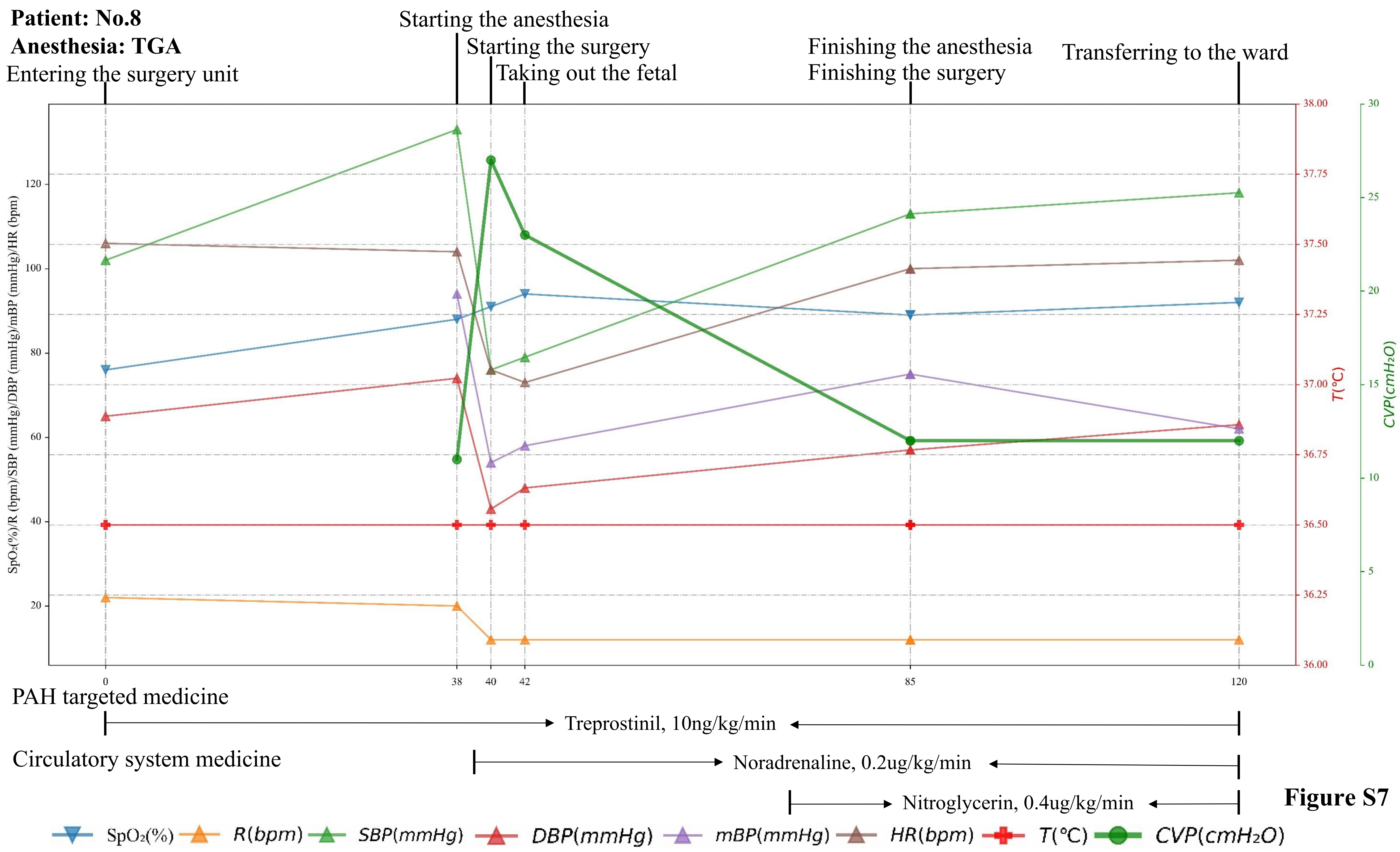

Patient: No.8
Anesthesia: TGA
Entering the surgery unit
Starting the anesthesia
Starting the surgery
Taking out the fetal
Finishing the anesthesia
Finishing the surgery
Transferring to the ward
SpO2(%)/R (bpm)/SBP (mmHg)/DBP (mmHg)/mBP (mmHg)/HR (bpm)
T(°C)
CVP(cmH2O)
PAH targeted medicine
Treprostinil, 10ng/kg/min
Circulatory system medicine
Noradrenaline, 0.2ug/kg/min
Nitroglycerin, 0.4ug/kg/min
SpO2(%)
R(bpm)
SBP(mmHg)
DBP(mmHg)
mBP(mmHg)
HR(bpm)
T(°C)
CVP(cmH2O)

Figure S7

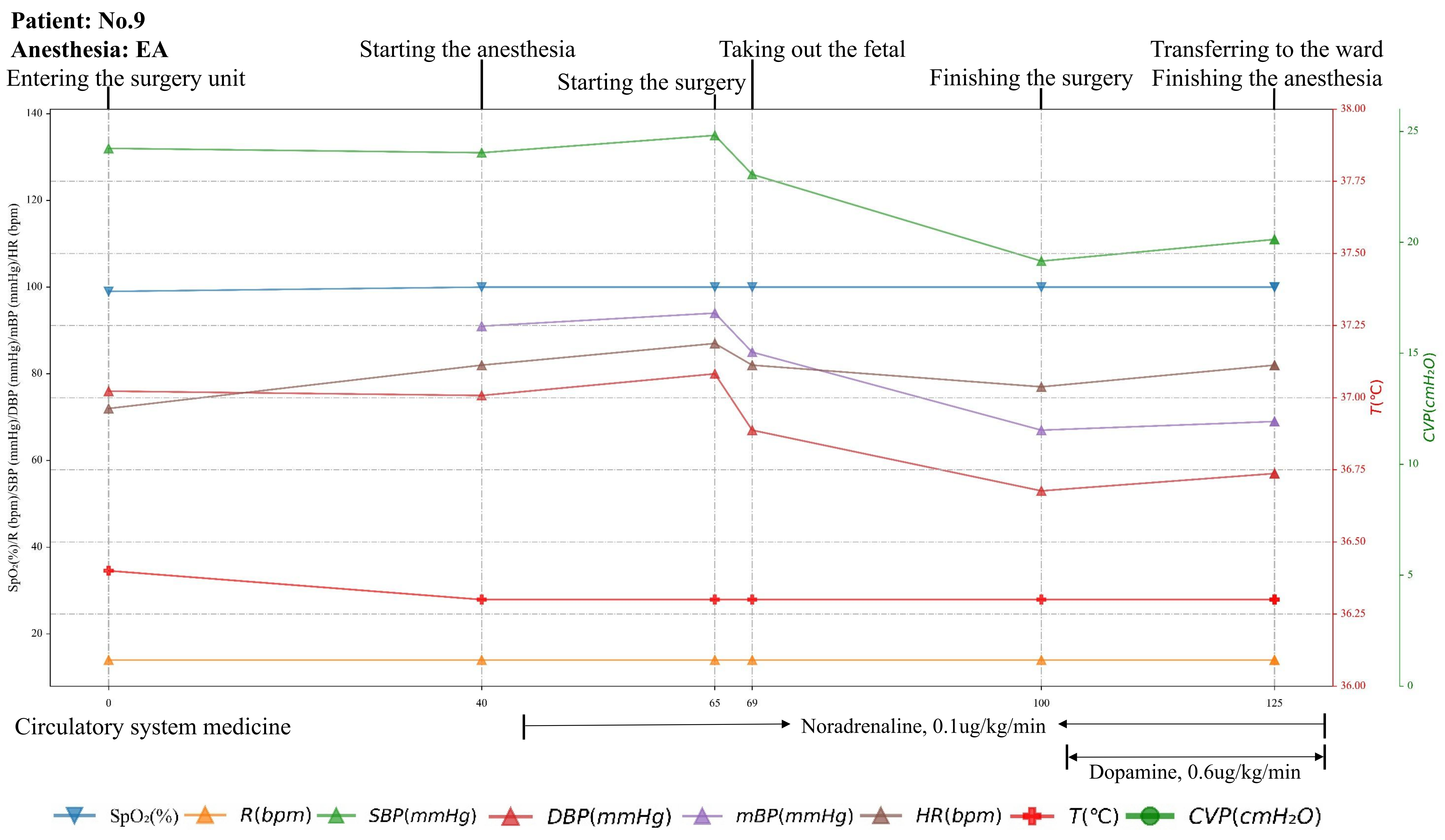

Patient: No.9
Anesthesia: EA
Entering the surgery unit
Starting the anesthesia
Starting the surgery
Taking out the fetal
Finishing the surgery
Transferring to the ward
Finishing the anesthesia
SpO₂(%)/R (bpm)/SBP (mmHg)/DBP (mmHg)/mBP (mmHg)/HR (bpm)
T(°C)
CVP(cmH₂O)
0
40
65
69
100
125
Circulatory system medicine
Noradrenaline, 0.1ug/kg/min
Dopamine, 0.6ug/kg/min
SpO₂(%)
R(bpm)
SBP(mmHg)
DBP(mmHg)
mBP(mmHg)
HR(bpm)
T(°C)
CVP(cmH₂O)

Figure S8

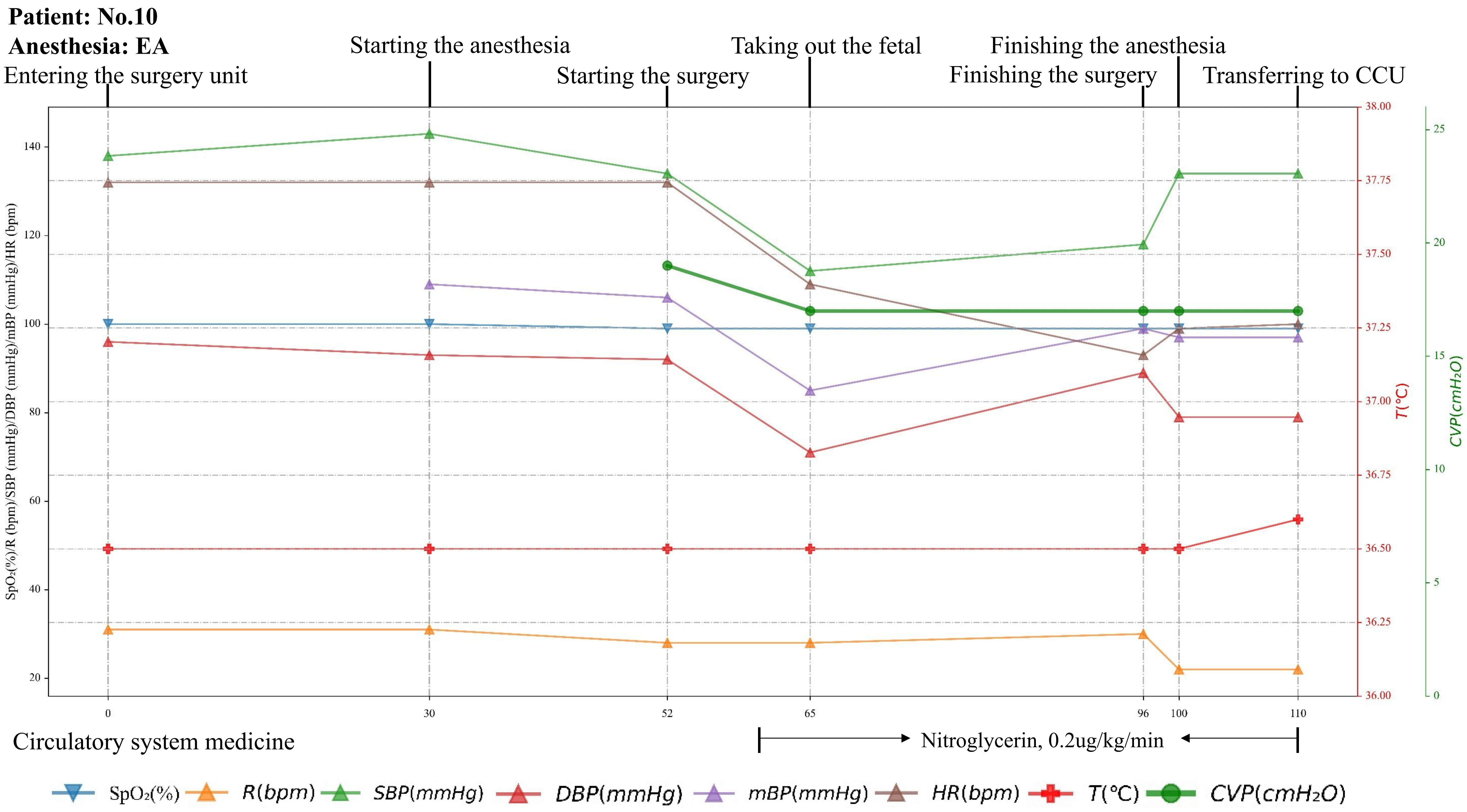
Patient: No.10
Anesthesia: EA
Entering the surgery unit
Starting the anesthesia
Starting the surgery
Taking out the fetal
Finishing the anesthesia
Finishing the surgery
Transferring to CCU
SpO₂(%)/R (bpm)/SBP (mmHg)/DBP (mmHg)/mBP (mmHg)/HR (bpm)
T(°C)
CVP(cmH₂O)
Circulatory system medicine
Nitroglycerin, 0.2ug/kg/min
SpO₂(%)
R(bpm)
SBP(mmHg)
DBP(mmHg)
mBP(mmHg)
HR(bpm)
T(°C)
CVP(cmH₂O)

Figure S9

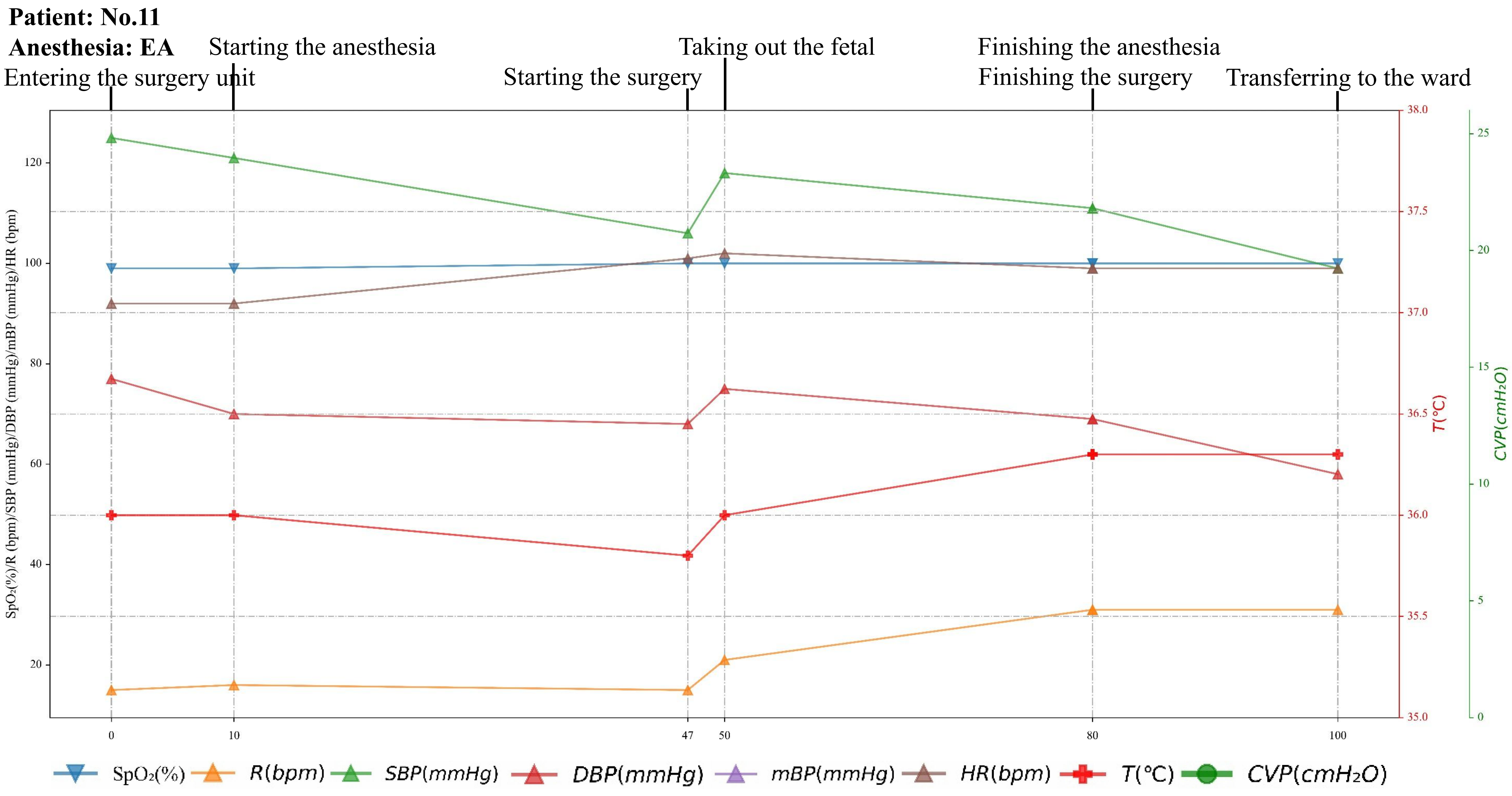

Patient: No.11
Anesthesia: EA
Starting the anesthesia
Entering the surgery unit
Starting the surgery
Taking out the fetal
Finishing the anesthesia
Finishing the surgery
Transferring to the ward
SpO2(%)/R (bpm)/SBP (mmHg)/DBP (mmHg)/mBP (mmHg)/HR (bpm)
T(°C)
CVP(cmH2O)
SpO2(%)
R(bpm)
SBP(mmHg)
DBP(mmHg)
mBP(mmHg)
HR(bpm)
T(°C)
CVP(cmH2O)

Figure S10

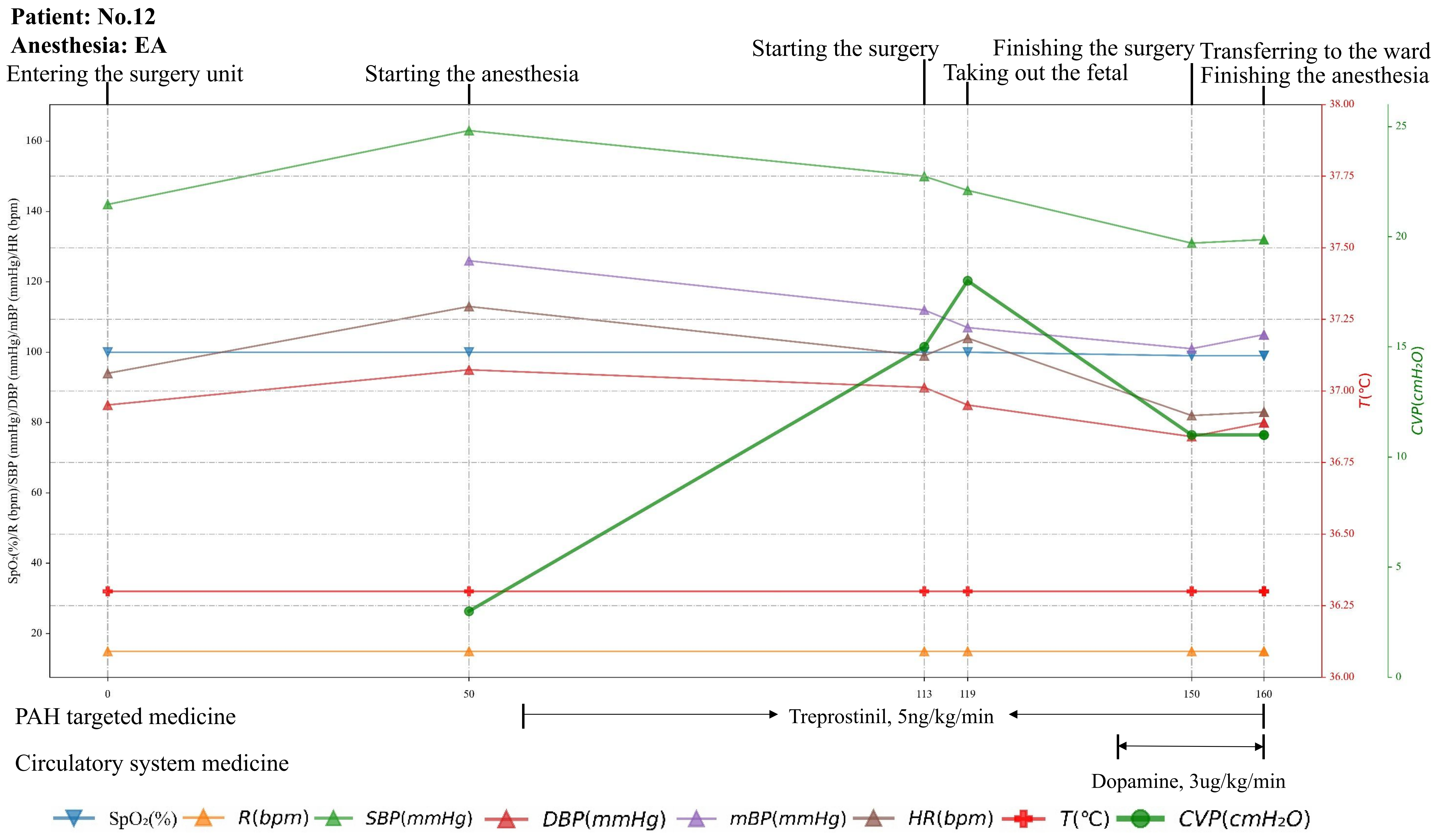

Patient: No.12
Anesthesia: EA
Entering the surgery unit
Starting the anesthesia
Starting the surgery
Taking out the fetal
Finishing the surgery
Transferring to the ward
Finishing the anesthesia
SpO2(%)/R (bpm)/SBP (mmHg)/DBP (mmHg)/mBP (mmHg)/HR (bpm)
T(°C)
CVP(cmH2O)
0
50
113
119
150
160
PAH targeted medicine
Treprostinil, 5ng/kg/min
Circulatory system medicine
Dopamine, 3ug/kg/min
SpO2(%) R(bpm) SBP(mmHg) DBP(mmHg) mBP(mmHg) HR(bpm) T(°C) CVP(cmH2O)

Figure S11

Patient: No.13
Anesthesia: EA

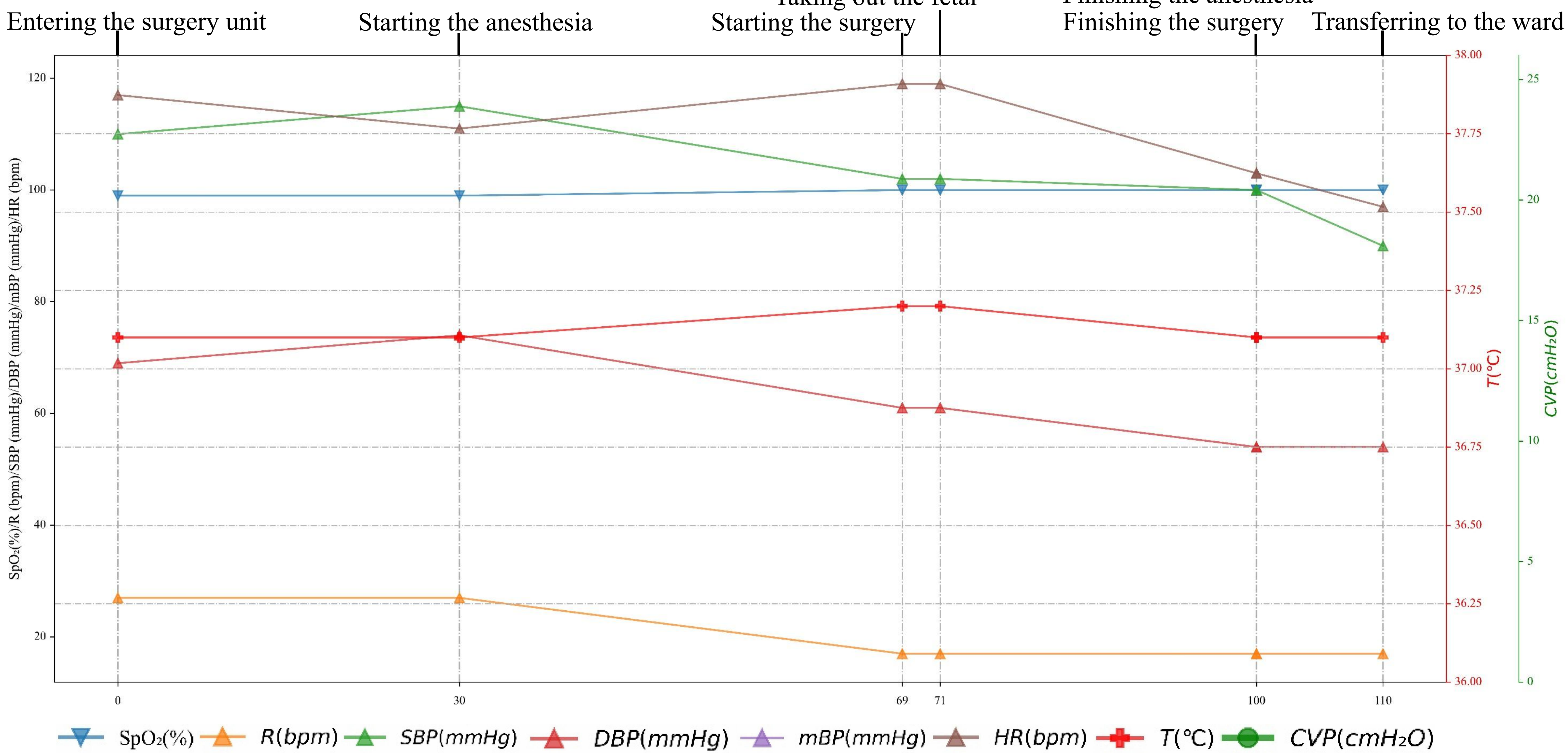

Figure S12

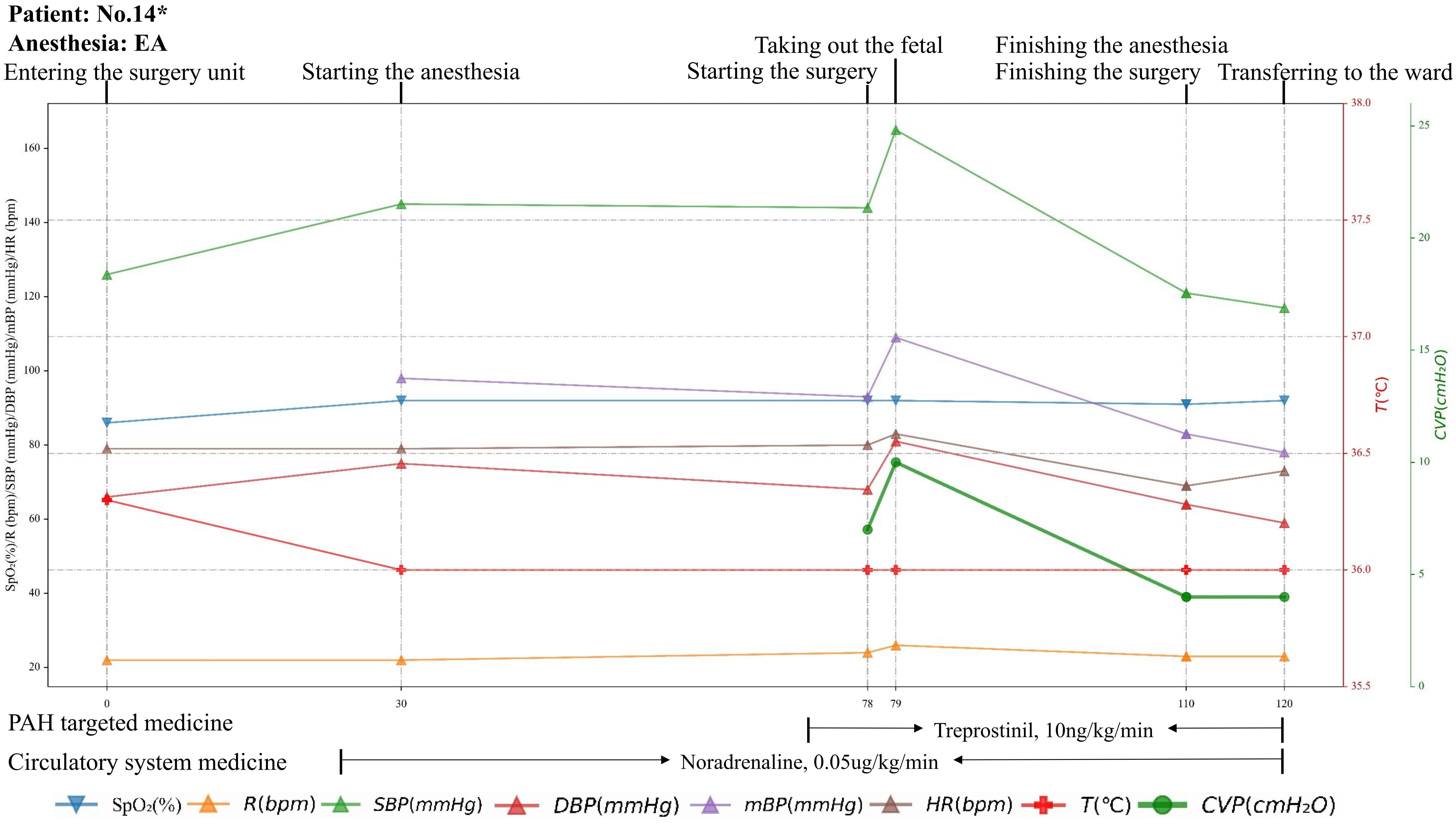
Patient: No.14*
Anesthesia: EA
Entering the surgery unit
Starting the anesthesia
Taking out the fetal
Starting the surgery
Finishing the anesthesia
Finishing the surgery
Transferring to the ward
SpO2(%)/R (bpm)/SBP (mmHg)/DBP (mmHg)/mBP (mmHg)/HR (bpm)
T(°C)
CVP(cmH2O)
PAH targeted medicine
Treprostinil, 10ng/kg/min
Circulatory system medicine
Noradrenaline, 0.05ug/kg/min
SpO2(%) R(bpm) SBP(mmHg) DBP(mmHg) mBP(mmHg) HR(bpm) T(°C) CVP(cmH2O)

Figure S13

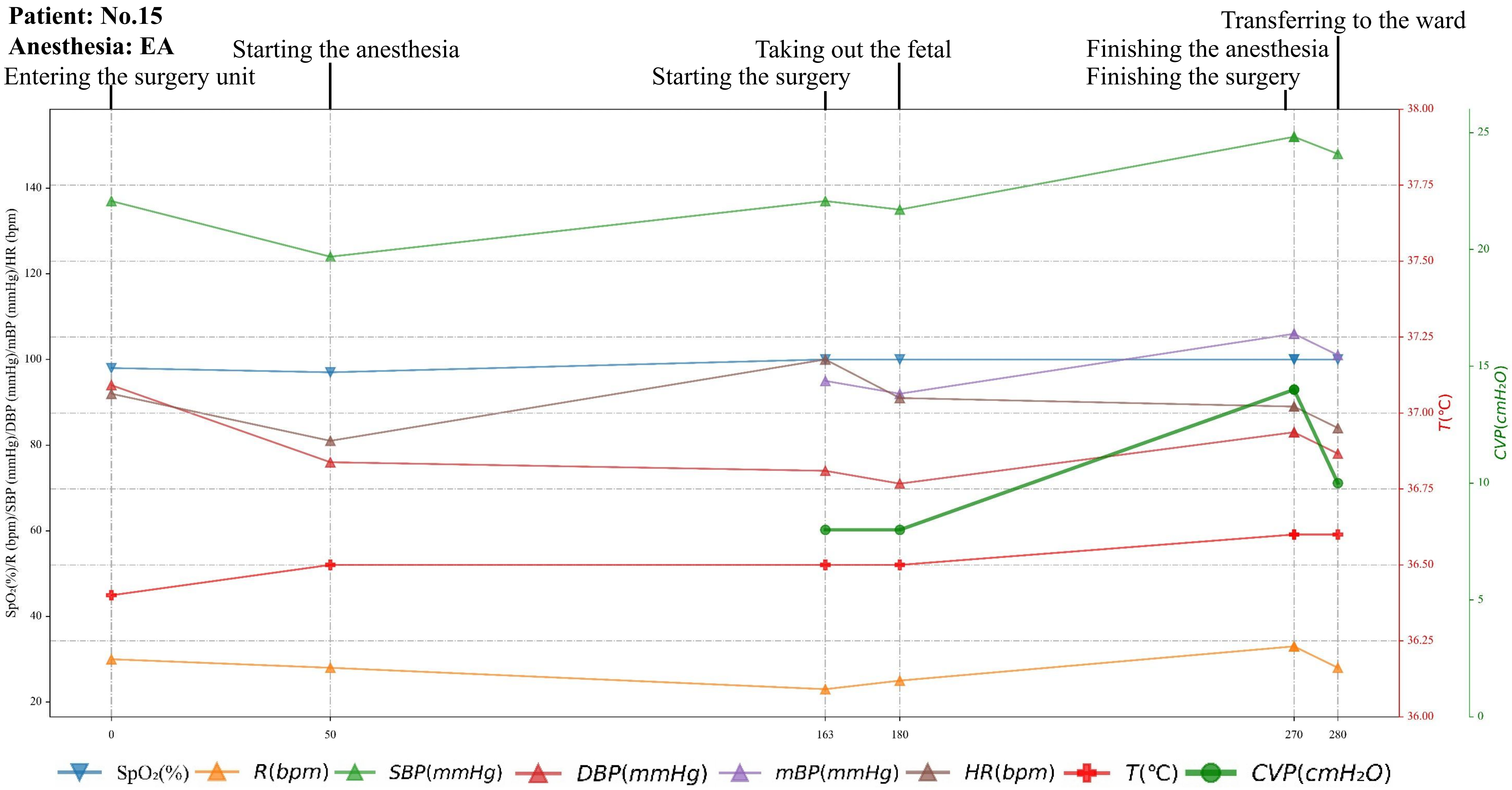

Patient: No.15
Anesthesia: EA
Entering the surgery unit
Starting the anesthesia
Starting the surgery
Taking out the fetal
Finishing the anesthesia
Finishing the surgery
Transferring to the ward
SpO2(%)/R (bpm)/SBP (mmHg)/DBP (mmHg)/mBP (mmHg)/HR (bpm)
T(°C)
CVP(cmH2O)
SpO2(%)
R(bpm)
SBP(mmHg)
DBP(mmHg)
mBP(mmHg)
HR(bpm)
T(°C)
CVP(cmH2O)

Figure S14

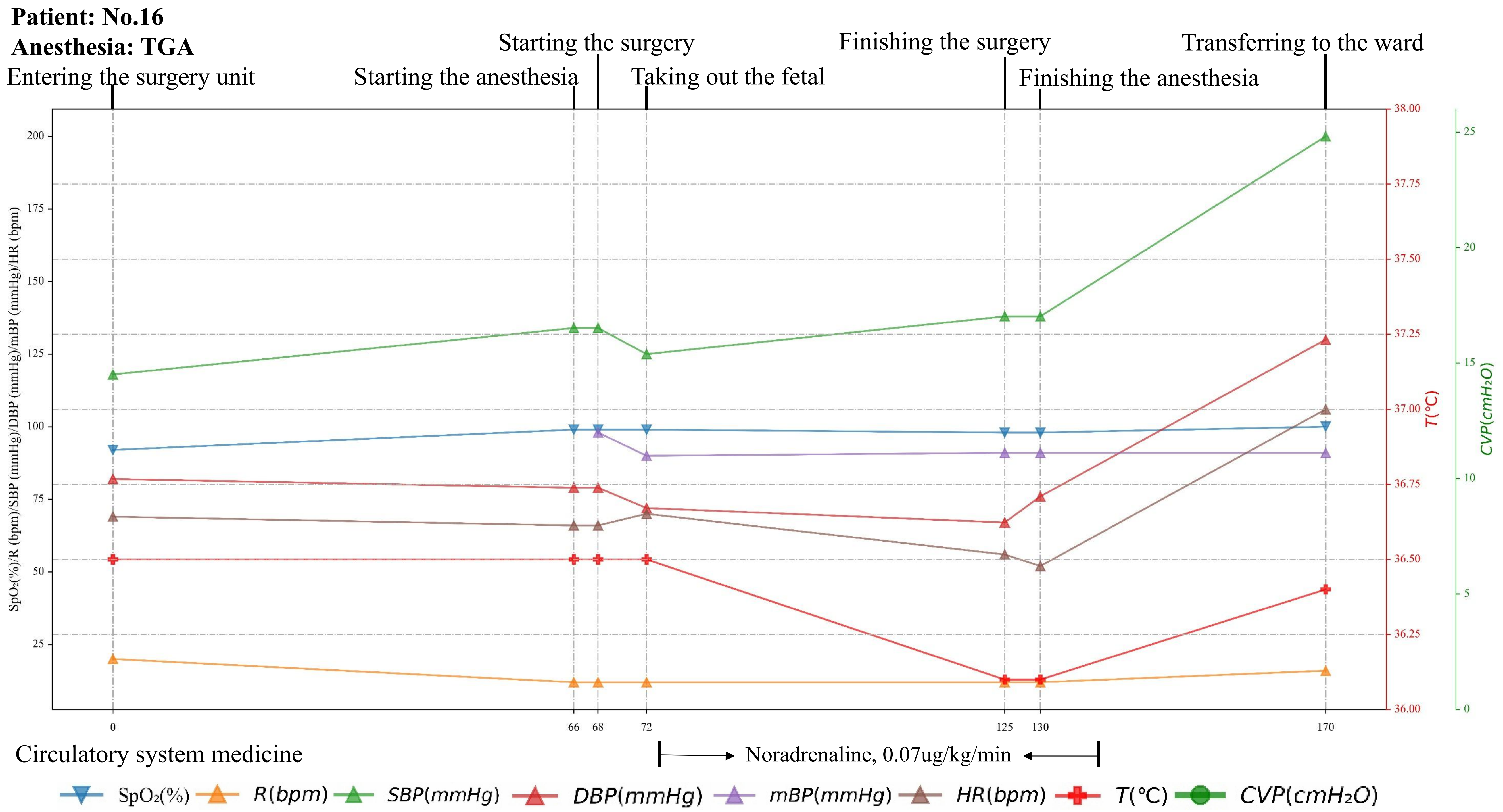
Patient: No.16
Anesthesia: TGA
Entering the surgery unit
Starting the anesthesia
Starting the surgery
Taking out the fetal
Finishing the surgery
Finishing the anesthesia
Transferring to the ward
SpO₂(%)/R (bpm)/SBP (mmHg)/DBP (mmHg)/mBP (mmHg)/HR (bpm)
T(°C)
CVP(cmH₂O)
Circulatory system medicine
Noradrenaline, 0.07ug/kg/min
SpO₂(%)
R(bpm)
SBP(mmHg)
DBP(mmHg)
mBP(mmHg)
HR(bpm)
T(°C)
CVP(cmH₂O)

Figure S15

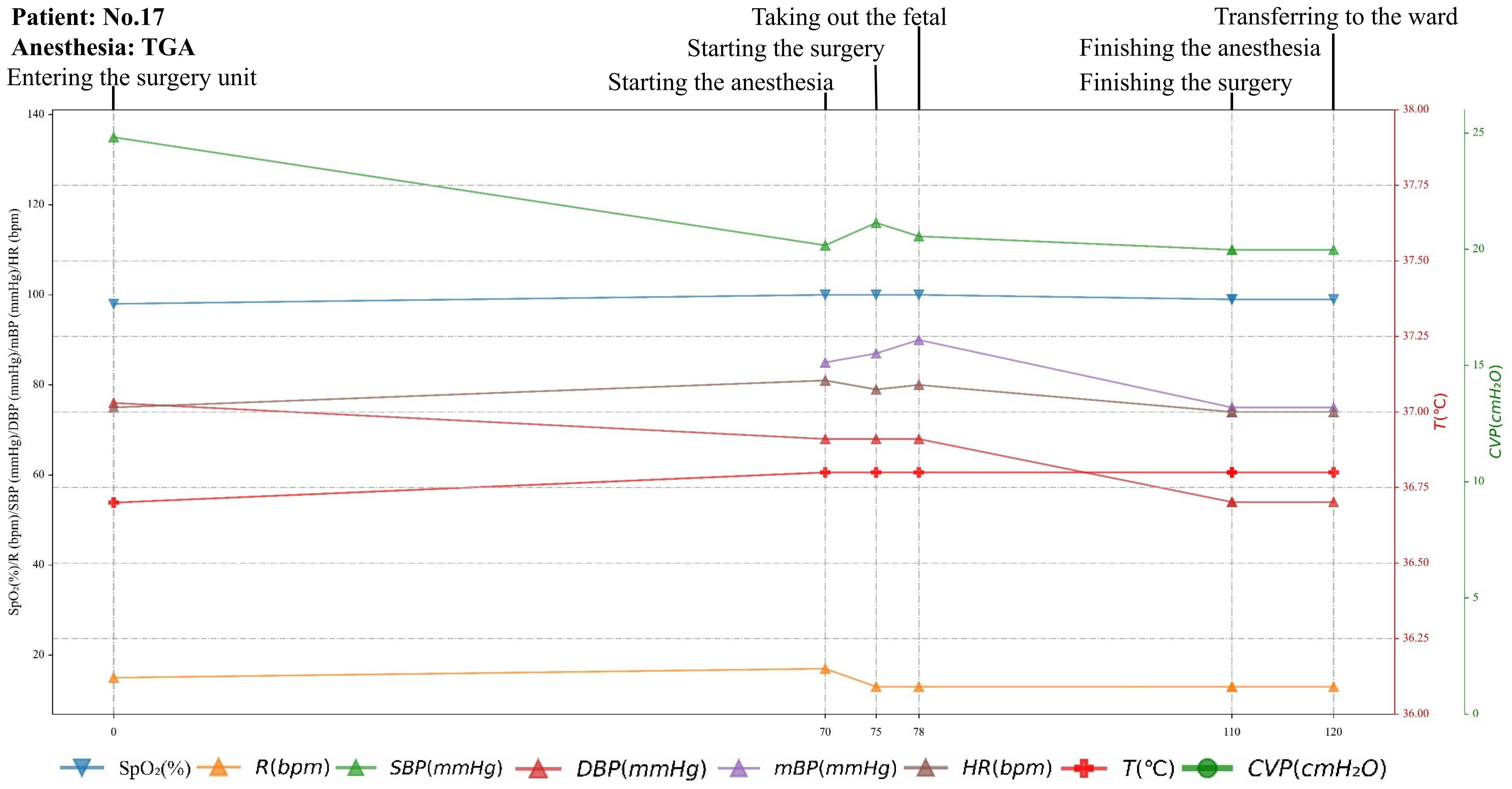

Patient: No.17
Anesthesia: TGA
Entering the surgery unit
Starting the anesthesia
Starting the surgery
Taking out the fetal
Finishing the surgery
Finishing the anesthesia
Transferring to the ward
SpO₂(%)/R (bpm)/SBP (mmHg)/DBP (mmHg)/mBP (mmHg)/HR (bpm)
T(°C)
CVP(cmH₂O)
SpO₂(%)
R(bpm)
SBP(mmHg)
DBP(mmHg)
mBP(mmHg)
HR(bpm)
T(°C)
CVP(cmH₂O)

Figure S16

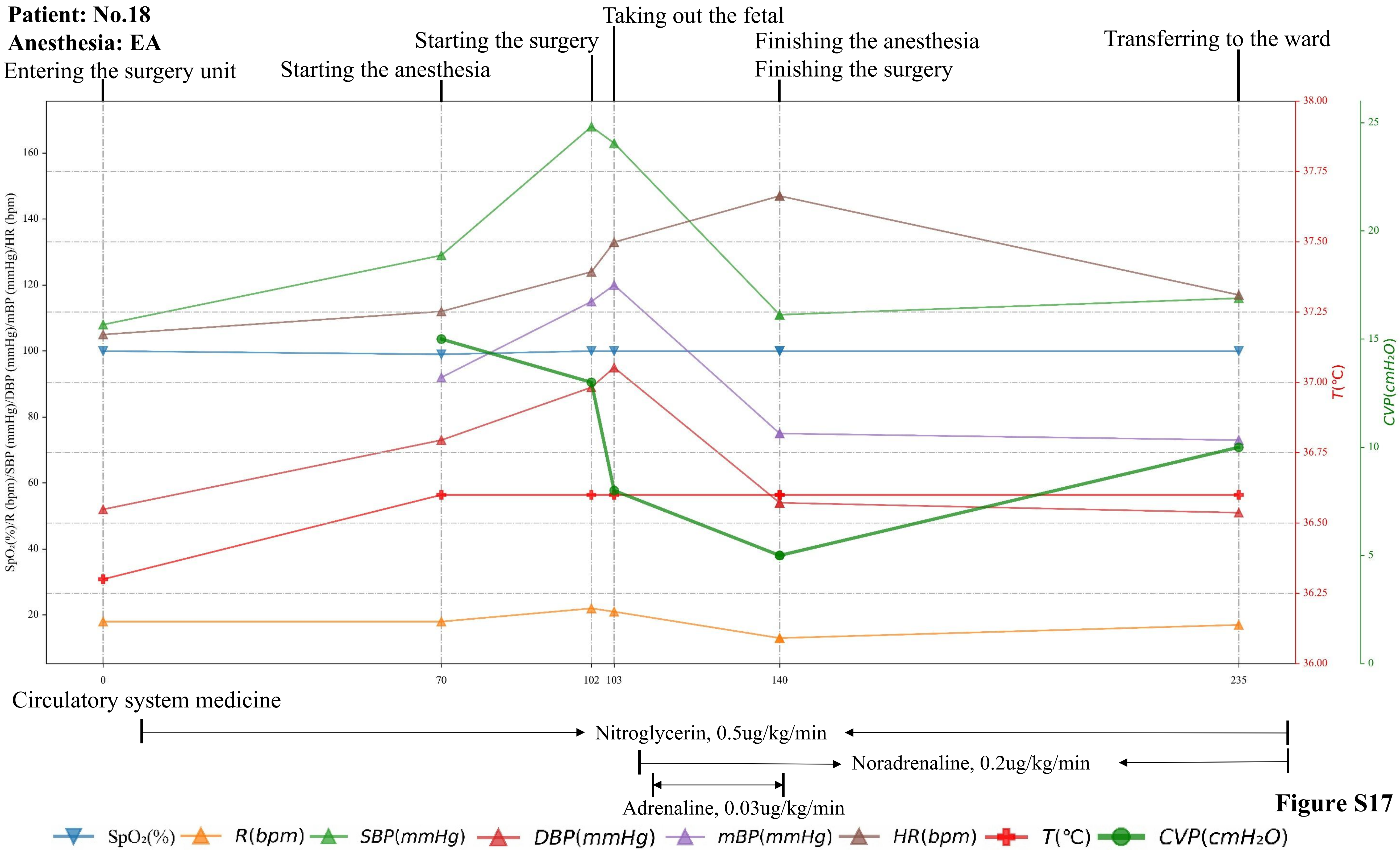
Patient: No.18
Anesthesia: EA
Entering the surgery unit
Starting the anesthesia
Starting the surgery
Taking out the fetal
Finishing the anesthesia
Finishing the surgery
Transferring to the ward
SpO₂(%)/R (bpm)/SBP (mmHg)/DBP (mmHg)/mBP (mmHg)/HR (bpm)
T(°C)
CVP(cmH₂O)
0
70
102
103
140
235
Circulatory system medicine
Nitroglycerin, 0.5ug/kg/min
Noradrenaline, 0.2ug/kg/min
Adrenaline, 0.03ug/kg/min
SpO₂(%)
R(bpm)
SBP(mmHg)
DBP(mmHg)
mBP(mmHg)
HR(bpm)
T(°C)
CVP(cmH₂O)

Figure S17

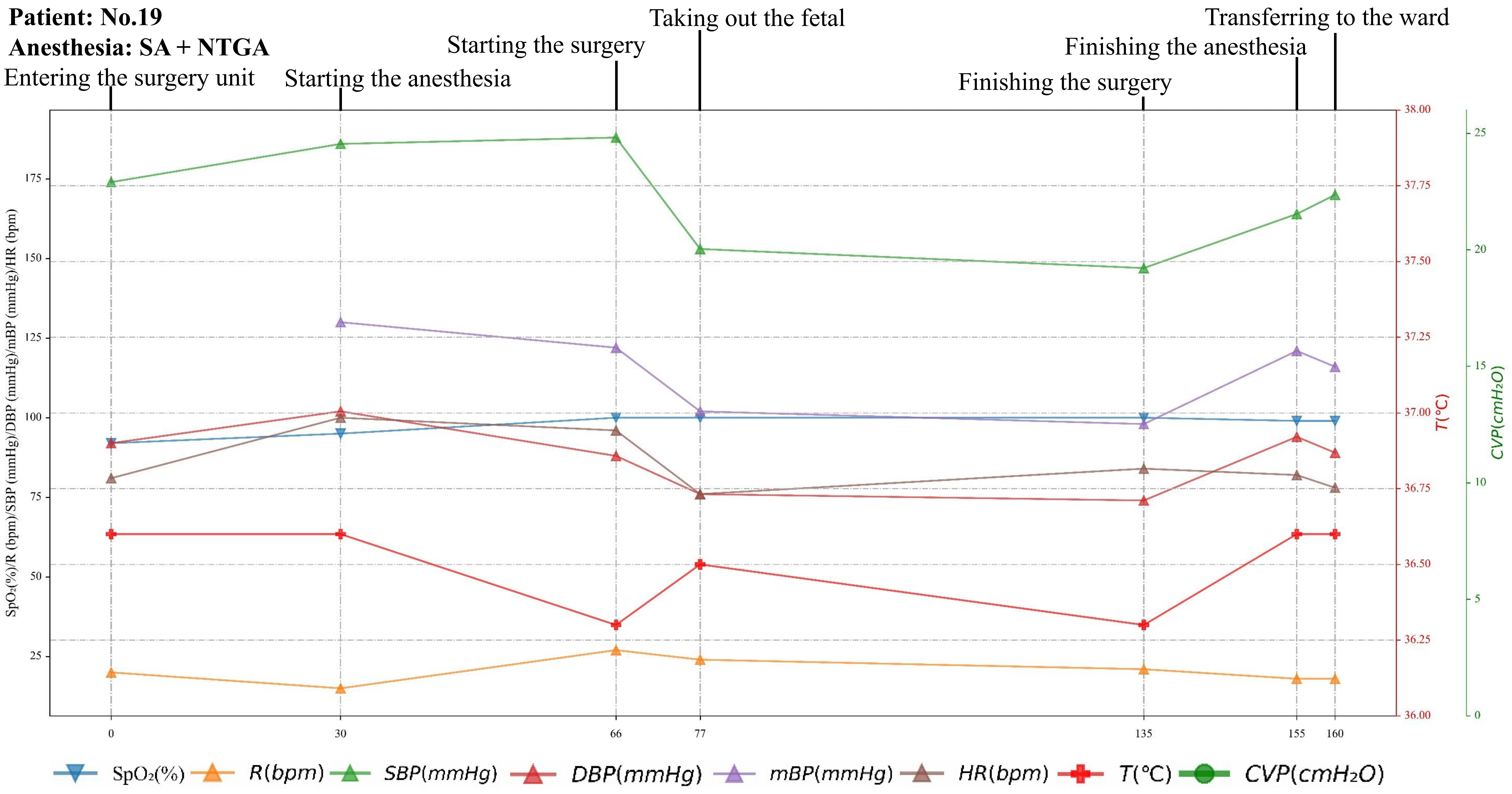

Patient: No.19
Anesthesia: SA + NTGA
Entering the surgery unit
Starting the anesthesia
Starting the surgery
Taking out the fetal
Finishing the surgery
Finishing the anesthesia
Transferring to the ward
SpO₂(%)/R (bpm)/SBP (mmHg)/DBP (mmHg)/mBP (mmHg)/HR (bpm)
T(°C)
CVP(cmH₂O)
0
30
66
77
135
155
160
SpO₂(%)
R(bpm)
SBP(mmHg)
DBP(mmHg)
mBP(mmHg)
HR(bpm)
T(°C)
CVP(cmH₂O)

Figure S18

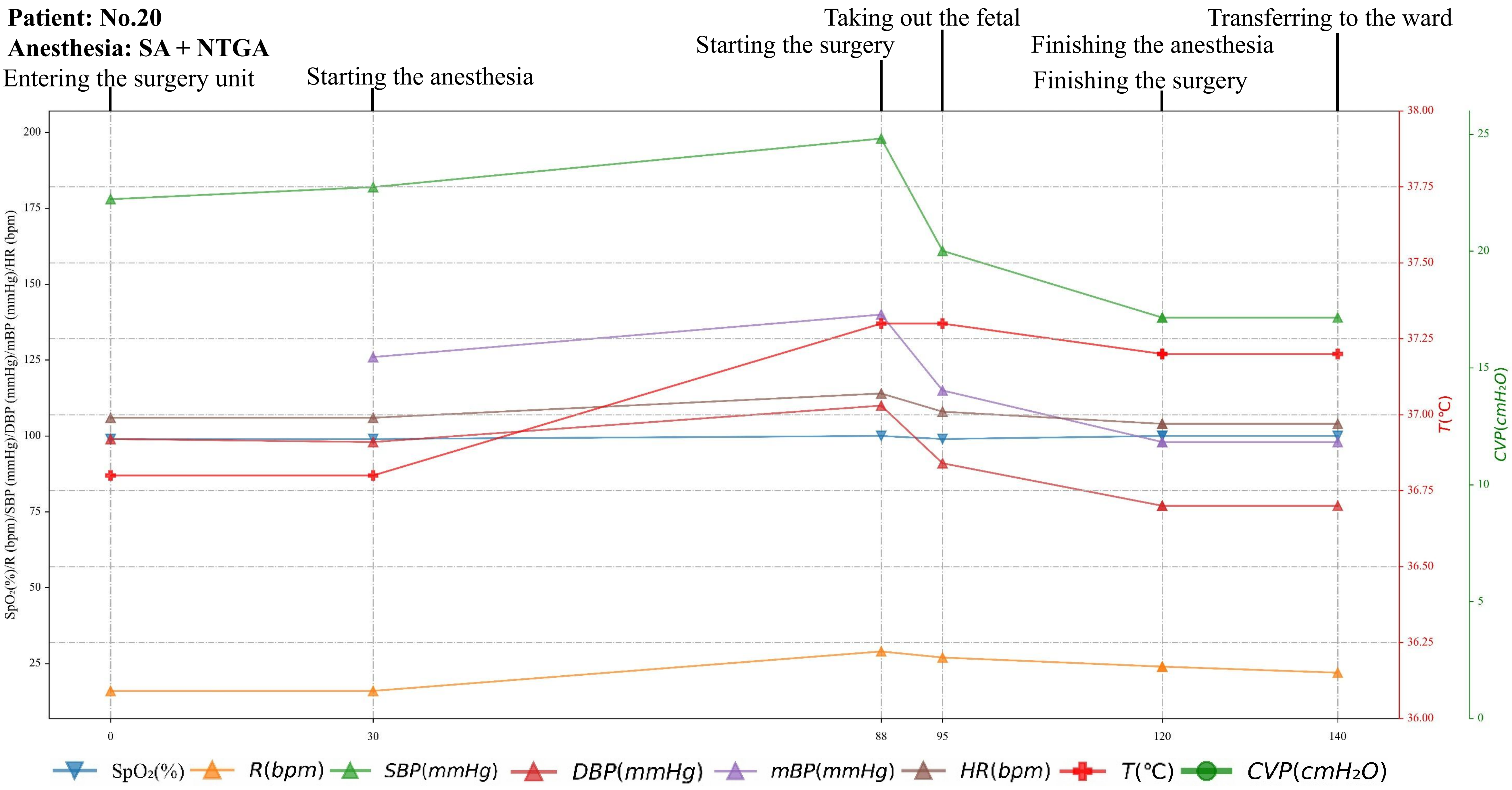
Patient: No.20
Anesthesia: SA + NTGA
Entering the surgery unit
Starting the anesthesia
Starting the surgery
Taking out the fetal
Finishing the anesthesia
Finishing the surgery
Transferring to the ward
SpO₂(%)/R (bpm)/SBP (mmHg)/DBP (mmHg)/mBP (mmHg)/HR (bpm)
T(°C)
CVP(cmH₂O)
SpO₂(%) R(bpm) SBP(mmHg) DBP(mmHg) mBP(mmHg) HR(bpm) T(°C) CVP(cmH₂O)

Figure S19

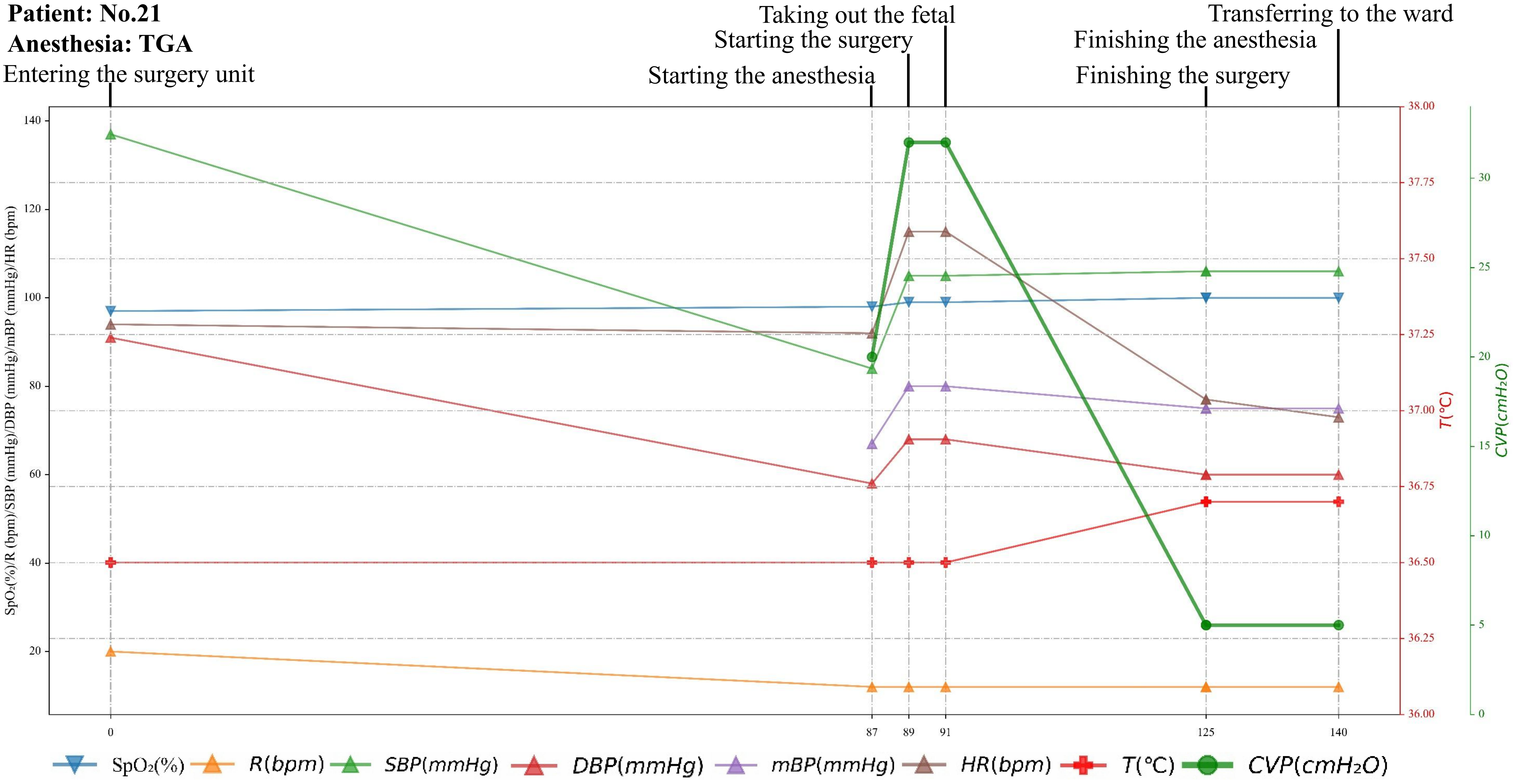

Patient: No.21
Anesthesia: TGA
Entering the surgery unit
Starting the anesthesia
Starting the surgery
Taking out the fetal
Finishing the surgery
Finishing the anesthesia
Transferring to the ward
SpO₂(%)/R (bpm)/SBP (mmHg)/DBP (mmHg)/mBP (mmHg)/HR (bpm)
T(°C)
CVP(cmH₂O)
0
87
89
91
125
140
SpO₂(%)
R(bpm)
SBP(mmHg)
DBP(mmHg)
mBP(mmHg)
HR(bpm)
T(°C)
CVP(cmH₂O)

Figure S20

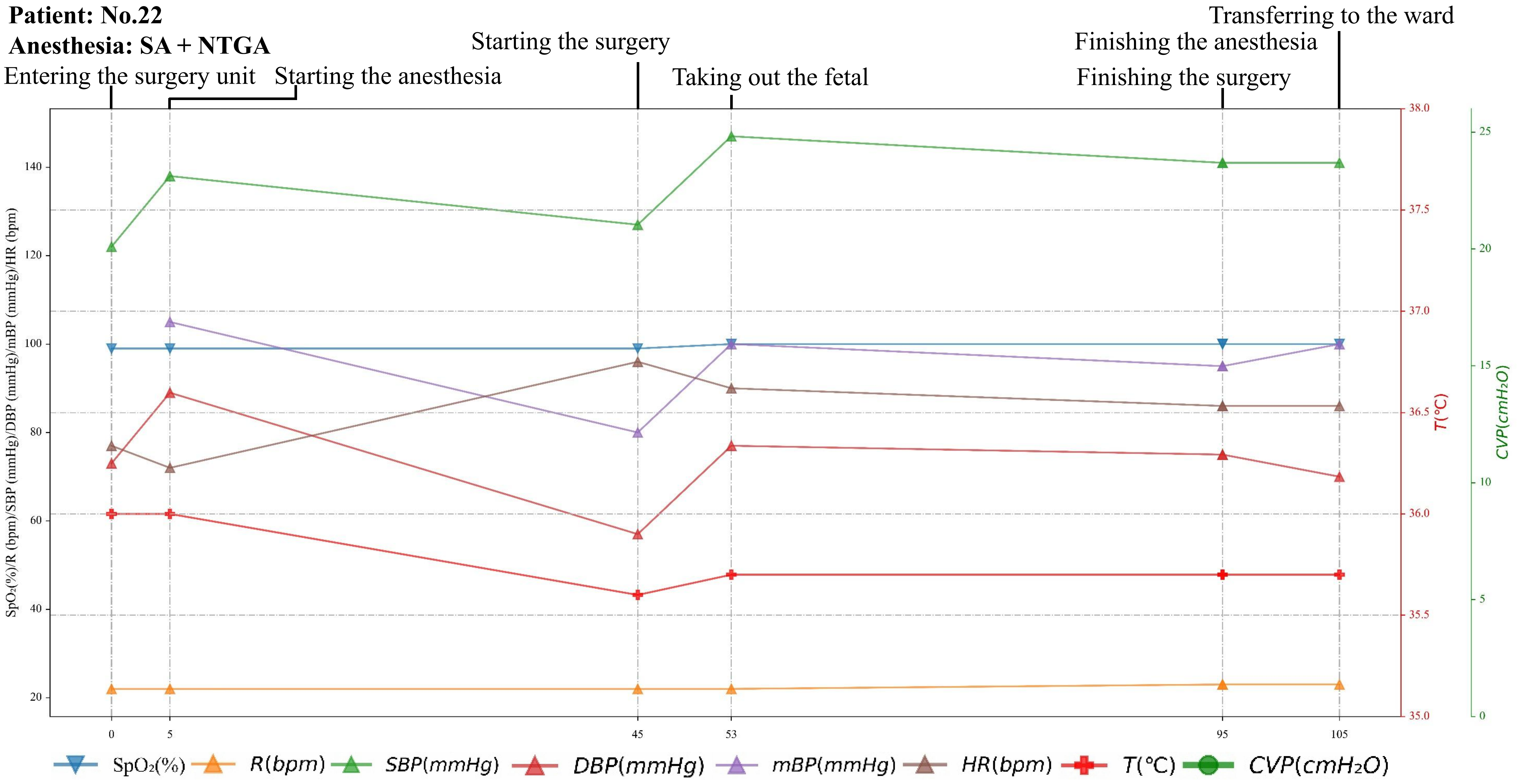
Patient: No.22
Anesthesia: SA + NTGA
Entering the surgery unit
Starting the anesthesia
Starting the surgery
Taking out the fetal
Finishing the anesthesia
Finishing the surgery
Transferring to the ward
SpO2(%)/R (bpm)/SBP (mmHg)/DBP (mmHg)/mBP (mmHg)/HR (bpm)
T(°C)
CVP(cmH2O)
SpO2(%) R(bpm) SBP(mmHg) DBP(mmHg) mBP(mmHg) HR(bpm) T(°C) CVP(cmH2O)

Figure S21
